# *Post hoc* experimental designs improve genetic trial analyses: A case study of cherrybark oak (*Quercus pagoda* Raf.) genetic evaluation in the western Gulf region, USA

**DOI:** 10.1371/journal.pone.0285150

**Published:** 2023-05-12

**Authors:** Chen Ding, Yuhui Weng, Tom D. Byram, Benjamin D. Bartlett, Earl M. Raley

**Affiliations:** 1 Western Gulf Forest Tree Improvement Program, Texas A&M Forest Service, Texas A&M University System, College Station, Texas, United States of America; 2 College of Forestry, Wildlife and Environment, Auburn University, Auburn, Alabama, United States of America; 3 Arthur Temple College of Forestry and Agriculture, Stephen F. Austin State University, Nacogdoches, Texas, United States of America; Austrian Federal Research Centre for Forests BFW, AUSTRIA

## Abstract

Oaks (*Quercus* spp.) are widespread hardwood trees in the Northern Hemisphere and of high ecological, economic, and social values. Optimal experimental design of genetic trials is essential for accurate estimates of genetic parameters and improving the genetic merit of breeding stock. Here, we evaluate the use of *post hoc* row-column factors combined with spatial adjustment to improve genetic analyses of parents and individual trees in field progeny tests of plantation hardwoods, using cherrybark oak (*Quercus pagoda* Raf.) as an example. For tree height, *post hoc* incomplete blocking reduced ~14% more of the within-block environmental variance compared to the randomized complete block design (RCBD) model. Incomplete blocking also improved the heritability estimates for height by 7% to 14% compared to the original RCBD model. No clinal trend for growth breeding values was identified due to provenances. Our approach warrants the initial selection for height as early as age ~10 based on its moderate narrow-sense heritability of 0.2; however, diameter and volume need longer evaluation times. The *post hoc* incomplete blocking is more efficient and promising to improve the genetic analysis of *Q*. *pagoda* to minimize the environmental heterogeneity influences. Adjusting competition and spatial effects, including the distance principal components and autoregressive residual structure notably improves the model fit based on the observed reductions in AICs and BICs. Employing our approach is promising for hardwood genetic improvement in the southern USA.

## Introduction

Oaks (*Quercus spp*.) are critical forest resources for the natural environment, society, and cultural heritage of human beings throughout Euro-Asia and America [1]. The oak species successfully form various hardwood forests from tropical to temperate zones and are abundant in both natural and plantation forests [2]. The success reforestation of oaks requires both efforts from the tree improvement and silvicultural prescriptions to promote growth, wood production, and urban forestry [3–5]. However, knowledge of quantitative genetics of growth, wood quality, and adaptation are limited for foresters among various oak species. The typical forest field testing for tree improvement was well documented and practiced for commercial tree species, e.g., pines and spruces, but not for oak species globally and regionally.

*Quercus pagoda Raf*. is a highly valued bottomland hardwood tree species in the southern USA with important ecological, recreation, landscaping, and economic values [6, 7]. *Q*. *pagoda* is a typical canopy- dominant and later successional species in natural stands with a high branch to stem density ratio [8] and has excellent lumber quality. Typical Q. pagoda trees reache 4-6m in height at ages 5–8 [6], while acorn production happens at ~age 15, which is considered as the selection age for growth [9]. Previous studies have shown that *Q*. *pagoda* along with other American oaks *Q*. *alba*, *Q*. *rubra*, *Q*. *falcata* express substantial phenotypic variation in height and diameter growth, crown form, and phenology within and between progenies raised in common garden studies [10]. Larger seedlings have a superior size in both root and shoot growth that is positively linked to the survival in reforestation [11]. Artificial and natural regeneration of *Quercus pagoda* is problematic and difficult as the result of many factors including the species competition, flood disturbances, shade sensitivity, gap sizes, seed dormancy, herbivory, and canopy-forest floor microenvironment [12–17]. There are also difficulties in precisely estimating the genetic parameters and merits in the field trials due to spatial competition for resources. Previous studies demonstrated the lower light availability limits biomass distribution and growth [13].

Previous small scale studies demonstrated some information of the genetic architecture of the species to date. The local population showed better adaptation and growth in a regional study while seedling survival and early growth showed less intraspecific variation in the main habitat range in Mississippi [10, 11, 18]. The recommendation was made that seed should be collected from areas in west Mississippi for both superior height and superior diameter growth for deployment in western Kentucky and west Tennessee [9]. Furthermore, *Q*. *pagoda* exhibited moderate genetic controls for height, diameter at breast height (DBH), and volume growth (h^2^ = 0.2–0.4) at ages 10–15, with family heritability as high as 0.5–0.7 [9]. The improved stock outperformed wild plantation stock for survival and resprouting rates and resiliency to dieback in southern Arkansas [11].

The traditional randomized complete block design (RCBD) and incomplete block design (ICBD) have been frequently employed as the standard designs in progeny testing due to their simplicity and robustness for exploiting genetic variability [19–21]. For RCBD in forest genetic tests, due to the high within-block environmental heterogeneity in blocks (~10-~30m in width or length depending on the species) local layout, as well as spatial dependency, the within-site, and within-block variations are difficult to dissect in conventional complete blocking [22]. Thus, the assumption of homogeneity in environment within a block is frequently violated due to micro-site, spatial autocorrelation, competition, and heterogeneity due to the block size.

The spatial related within-site environmental variations can be continuous [23] or discontinuous depending on the trial condition and design. Continuous variation follows the geographic or edaphic gradients such as topography, slope, aspect, or soil moisture [24]. And discontinuous variation is frequently associated with patchy fertilization, low spots with excessive moisture, random soil conditions including rocks and sands, uneven management, or irregular shapes of blocks. Other factors include mechanical and chemical preparation of the plantation site, seedlings, and planting methods [24].

To improve the genetic parameter estimates by reducing the spatial and environmental variation on micro-site and within replicates, various approaches have been developed at both the design and statistical analytics stages including, row-column [25], spatial analyses [26], and *post hoc* blocking in both forestry and crop sciences [24, 27, 28]. The *post hoc* adjustment method uses the existing design information more efficiently to improve the accuracy and reliability of genetic parameters of tested materials [24, 27], by adjusting the within-site variation and random environmental noises.

Competition as a typical ‘noise’ in the genetic trials causes the within-site variation [23, 29], along with other site-specific factors including tree-to-tree interaction [30], heterogeneous soil fertility and moisture regimes [31, 32], invasive and non-test vegetation (e.g., herbaceous and shade-tolerant shrubs), insects or diseases, as well as other silvicultural aspects [23]. The spatial arrangement and patterns of individually tested trees indicate the neighboring competition that can be modelled by spatial autocorrelation and heterogeneous variation, especially for older progeny trials [23]. Previous research on *post hoc* experiments utilized the within-block effect to adjust the competition from neighboring trees [33]. *Post hoc* analyses and improved experimental designs offer advanced analytical tools that better control inter and intra block environment variations in the genetic trials and improve the breeding and selection results [28, 34].

In forest genetic trials, the spatial adjustment has been applied in the RCBD and ICBD designs to dissect the spatially correlated variation out of the random environment residual [35, 36]. In recent decades, row-column and incomplete blocking were evaluated and employed in genetic trials of multiple commercial tree species [22, 27, 37], grass [28], and crop species [38] to account for the heterogeneity of environmental variances. Incorporating spatial autocorrelation accounts for the spatial dependence in the covariance structure and decouples the effect of the local environment in the linear mixed effect model [19, 26, 39–45]. Unlike conifer genetic tests, hardwood trees such as *Quercus pagoda* Raf. require greater spacing for crown development and radial growth, allowing for greater environmental variation; thus, modeling with spatial effect, row-column, and incomplete blocking are promising for improving the genetic parameter estimates in this species.

The Western Gulf Forest Tree Improvement Program (WGFTIP) of the Texas A&M Forest Service has the only region-wide breeding program for *Q*. *pagoda*, focusing its efforts on improving volume production. In this study, we used WGFTIP *Quercus pagoda* Raf. progeny tests to evaluate the *post hoc* adjustment of row-column factors in field progeny tests originally established utilizing RCBD. Here we tested the *post hoc* treatments incorporated with spatial analyses to account for a more heterogeneous variance structure compared to traditional RCBD testing. We hypothesis that the *post hoc* method increases the signal-to-noise ratio for the genetic variances and improves genetic analyses of RCBD tests. Furthermore, we will assess the genotype-by-environment (GXE) interaction for growth in the region.

## Materials and methods

### Progeny testing and plant materials

**Fig 1:**
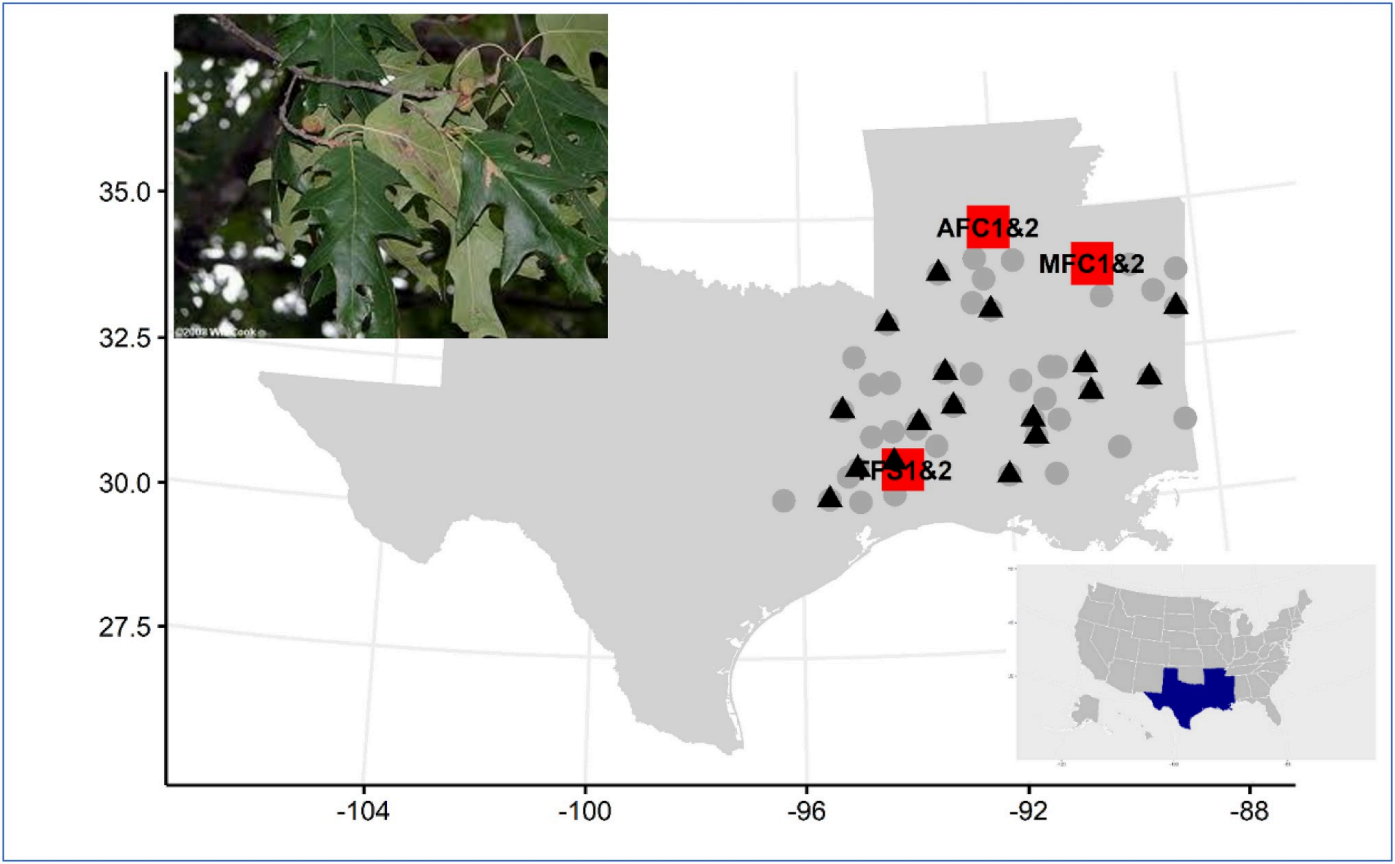
Map of the WGFTIP *Q*. *pagoda* Raf. progeny test sites and selection locations. Note, The two series of *Q*. *pagoda* Raf. progeny tests were planted at the adjacent locations in Arkansas and Mississippi. The Texas sites were established at separated locations for two series. The grey points depict the 305 1^st^-generation selections originating from 51 counties; the triangles represent the 52 2^nd^-generation selections from parents originating in17 of those 51 counties. The x-axis is the longitude and y-axis is the latitude. Map data were obtained from *urbnmapr* R package and state boundaries were from the US Census Bureau (www.census.gov).

The Western Gulf Forest Tree Improvement Program (WGFTIP) has operated an improvement program for *Q*. *pagoda* since the late 1970s. The first-generation population consisted of 305 selections made across 51 counties in four states: Texas (TX), Arkansas (AR), Louisiana (LA), and Mississippi (MS). These selections were evaluated for survival and growth in region-wide progeny tests from which 62 half-sib second-generation selections were made in the top 20% of the families (representing 17 of the original 51 counties) based on age-15 growth evaluations. The second-generation open-pollinated progenies were tested in two test series by the Texas A&M Forest Service (TFS), the Arkansas Forestry Commission (AFC), and the Mississippi Forestry Commission (MFC). (Fig 1). Six progeny trials were established in 2007 and 2008 and evaluated 52 of the 62 selections. Each test was established using a randomized complete block design (RCBD), with 30 blocks of single-tree plots planted on a 10’x10’ spacing (3.05m x 3.05m). At the AR and MS locations, series 2 tests were established adjacent to the series 1 trials. Age-10 growth data were collected on 4,553 individual trees. Tree volume (dm^3^) was calculated as 0.02618*DBH^2^*Height (Table 1). In our analyses, volume was transformed as a natural log (volume+0.1) to improve the model fit and optimize the initial values of the mixed model.

**Table 1:** WGFTIP *Q*. *pagoda* Raf. progeny test location and layout information and age-10 phenotypic means.

Trial	County, State	Latitude	Longitude	Year Planted	Blocks	Rows	Columns	Survival (%)	Height (m)	DBH (cm)	Volume (dm^3^)
*Series 1*											
AFC1	Pulaski, AR	34.76	-92.13	2007	30	25	48	72.3	8.87 (27.1)	10.33 (45.8)	34.91 (101.4)
MFC1	Tallahatchie, MS	34.02	-90.08	2007	30	40	30	90.3	8.59 (19.6)	9.50 (30.6)	24.12 (70.3)
TFS1	Jasper, TX	30.95	-94.13	2007	30	30	48	79.3	5.75 (43.6)	7.04 (59.3)	13.93 (133.3)
*Series 2*											
AFC2	Pulaski, AR	34.76	-92.13	2008	30	20	60	93.9	6.41 (28.8)	7.39 (47.4)	13.35 (107.5)
MFC2	Tallahatchie, MS	34.02	-90.08	2008	30	25	30	64.1	5.79 (30.9)	6.61 (44.5)	9.55 (101.7)
TFS2	Jasper, TX	30.70	-94.05	2008	30	25	30	62.3	8.27 (27.1)	11.23 (35.1)	34.11 (76.8)

Note: Series 1 and 2 tested 29–36 families per test, when total families including fillers reached 36 per test. There are 21 families tested (62%) in both Series 1 (32 families) and Series 2 (34 families). There are 52 unique selections tested in total. The values in parentheses are the coefficients of variation for height DBH, and volume. The planted tree number is 1200 for Series 1 and 750 for Series 2 per test. Each test contains 30 individuals per family. The row-column coordinates were assigned by the continuous identification numbers of row column blocks along the associated directions. The real coordinates were close to such proxy because of the equal distancing of individual trees in the RCBD design.

### Post hoc blocking and spatial analysis

We used modified-complete and incomplete (sub) *post hoc* blocking methods. Test layouts generally consisted of between four and six tiers of blocks running north to south and four to 10 blocks in the east-west direction. Continuous row and column coordinates were created based on the individual site map in each trial. We kept the rows in the direction of latitude (South->North) and the columns are from west to east or the reverse as east to west (based on the original layout sequence of blocks and tree marking sequence). All missing and filler trees were counted to create the complete trial map. From previous studies, complete individual tree grids benefit computational speed [23, 40]. For the modified-complete blocking, five to six ‘row blocks’ were delineated corresponding to the number of tiers in the test with five to six blocks included per ‘row block’; similarly, ‘column-blocks’ were delineated in the orthogonal direction for columns. Within the RCBD, tests generally had five to eight rows and five to eight columns per block. The incomplete block design grouped 25–60 rows and 5–6 trees per row into each incomplete block into a rectangular shape. This had the impact of changing the number of trees per block from 25 and 40 (series 2 and series 1, respectively), to between 125 and 360. The complete and incomplete blocking methods are shown in the S10 Fig. The concept of the *post hoc* blocking methods and example cases are demonstrated in [27].

The distance matrix for individual trees was calculated with the *dist()* function in R based on the distances between each pair of individual trees. A distance matrix was made based on the spatial coordinates of each tree, of which the dimension was #row x # column per site. We created a principal component analysis (PCA) derived distance index from the distance matrix by extracting the first three principal components as three neighboring distances to capture the variability due to spatial location and distance with the *prcomp()* function in R. The first to the third principal components (PC1, PC2, and PC3) were extracted for each tree based on the distance matrix. They summarized the overall spatial distances of individual trees. Based on the first three distance principal components, 98%-100% of the total environmental variance was explained.

The nearest neighbor competition index was categorized from 0 to 4 based on the radius of each individual (focal) tree to the most adjacent tree which was an approximation of neighbor interaction due to the spacing and location. We employed the distance matrix to identify the nearest neighbors at the one-unit radius due to the same spacing distance in the row and column direction of the trial as 3.05m. The number of adjacent trees within the radius was recorded for individual trees as the competition index: zero meant no close tree—the lowest competition level; and four indicated the highest competition with four trees nearby. The competition index was treated as a semi-categorical index. The competition condition varied by site and lower spatial heterogeneity was expected to enable the less biased estimation of competition [30, 33].

Acronyms for blocking, spatial and model factors are listed in Table 2.

**Table 2:** Scheme of sub-blocking model effects, variance-covariance structures, and spatial-statistic adjustment of y using multi-environmental trial (MET) analysis as an example.

Model type of MET	Fixed effects	Random effects
Sub-blocking methods 1–6		
1. Simple residual variance (*SUBBLOCKING*)	x (Row); y (Column); Tr;	a; ae; r; row; column; e; $R={\sigma_{\varepsilon }^{2}AR1+\sigma }_{e}^{2}⊗I$; $e\sim N(0,\;\sigma_{e}^{2}⊗I$*)*
2. Heterogeneous residual variance (*SUBHETERO*)	As previous;	Same terms but heterogeneous variance-covariance structure of random effect e, $R=\sigma_{\varepsilon }^{2}AR1+{⊕}_{i=1}^{i=n}{\sigma_{e}^{2}}_{i}$;
3. Nearest neighbor covariate with simple residual variance (*SUBNN*)	x (Row); y (Column); Tr; competition;	Same terms but simple variance-covariance structure e, $R={\sigma_{\varepsilon }^{2}AR1+\sigma }_{ei}^{2}⊗I$;
4. Nearest neighbor covariate with heterogeneous residual variance (*SUBNNHETERO*)	As above;	Same terms but heterogeneous variance-covariance structure of e, $R=\sigma_{\varepsilon }^{2}AR1+{⊕}_{i=1}^{i=n}{\sigma_{e}^{2}}_{i}$;
5. Nearest neighbor distance covariate with heterogeneous residual variance (*SUBNNPCA1*)	x (Row); y (Column); Tr; PC1-3;	Same as above;
6. Combined nearest neighbor distance covariate with simple residual variance (*SUBNNPCA2*)	x (Row); y (Column); Tr; competition; PC1-3;	Same terms but simple variance-covariance of e, $R={\sigma_{\varepsilon }^{2}AR1+\sigma }_{ei}^{2}⊗I;$
Complete blocking methods 7–12		
7. Simple residual variance (*BLOCKING*)	x (Row); y (Column); Tr;	a; ae; r; row_c; column_c; e, simple random residual variance-covariance structure; $R=\sigma_{\varepsilon }^{2}AR1+{⊕}_{i=1}^{i=n}{\sigma_{e}^{2}}_{i}$;
8. Heterogeneous residual variance *(BLKHETERO)*	As above;	Same terms as above but heterogeneous trial terms of random e, $R=\sigma_{\varepsilon }^{2}AR1+{⊕}_{i=1}^{i=n}{\sigma_{e}^{2}}_{i}$;
9. Nearest neighbor covariate with simple residual variance (*BLKNN)*	x (Row); y (Column); Tr; competition;	Same terms as above but simple trial terms of random effect e;
10. Nearest neighbor distance covariate with heterogeneous residual variance *(BLKNNHETERO)*	As above;	Same terms as above but heterogeneous trial terms of random effect e;
11. Nearest neighbor, combined covariate with heterogeneous residual variance (*NNPCA1)*	x (Row); y (Column); Tr; PC1-3;	Same as above;
12. Combined nearest neighbor and distance covariates with simple residual variance (*NNPCA2)*	x (Row); y (Column); Tr; competition; PC1-3;	Same terms as above but simple trial terms of random effect *e*.

Note, For the Sub-blocking methods, x (Row), x coordinates nested in row plot in the trial; y (column), y coordinates nested in column plot in the trial; Tr, trial effect; competition, competition index nested in trials; PC1-3, Principal components 1, 2, and 3 of neighbor distances nested in trials;

For the random effects, a, additive genetic effect; *a* ~ $N\left(0,\sigma_{A}^{2}⊗A\right)$; A is the kinship matrix; ae, simple genetic and trial interaction, *ae*~ $N\left(0,\sigma_{ae}^{2}⊗I\right)$; r, original replicate in trial; *r~* $N\left(0,\sigma_{r\left(t\right)}^{2}⊗I\right);\;$ row, sub- incomplete row block nested in block-trial; *row~$\;N\left(0,\sigma_{row(t)}^{2}⊗I\right)$*; column, sub-incomplete column block nested in block-trial; *column*~$\;N\left(0,I\sigma_{column(t)}^{2}⊗I\right)$; e, simple random residual effect, *e*~*MVN*(0,*R* ⊗ *I*), and the residual variance–covariance matrix of random residuals is *R*; *I* is the associated identify matrix;

The heterogeneous residual models use a block-diagonal variance-covariance structure to fit in order to adjust heterogeneity of trait variation among sites which as a residual variance for each site as following,

$R=\sigma_{\varepsilon }^{2}AR1+{\;⊕}_{i=1}^{i=n}{\sigma_{e}^{2}}_{i}$, ⊕ is the direct sum operator, and ${{\sigma_{\varepsilon }^{2}AR1+\sigma }_{e}^{2}}_{i}⊗I$ is the i^th^ site-specific residual variance among n sites [52];

For the complete blocking methods, row_c, complete row block nested in trial, *row_c*~$\;N\left(0,\sigma_{rowc(t)}^{2}⊗I\right)$; column_c, complete column block nested in trial, *column_c~$\;N\left(0,\sigma_{columnc(t)}^{2}⊗I\right)$; I* is the identity matrix for the row_c or column_c. Autoregressive residuals were applied for all models above.

### Linear mixed model analysis

Three *post hoc* treatment statistical analyses for predicting the breeding values of female families were carried out: (1) single-site analyses to estimate the breeding values which is shown in the appendix; (2) analyses of each series; (3) combined analyses of two series (MET). Single series analyses were not reported here due to their similar trends as in the MET and relatively higher standard errors of variance components for the same population tested at the same breeding regions.

The linear model of a multi-environment trial (MET) of growth traits is

$$Y=X\beta +Z_{1}a+Z_{2}ae+Z_{3}r+Z_{4}row+Z_{5}column+\;e$$ (1)

where, the fixed effect is **β** including the trial effect, row effect and column effect nested within the trial respectively; series and provenance effect were not fitted here but considered in the original randomized block design models (all random effects) without the *post hoc* information; *a* denotes the random vector of additive genetic effect, with a $\sim N(0,\sigma_{A}^{2}A)$, where $\sigma_{A}^{2}$ is the additive genetic variance and *A* is the pedigree kinship matrix; *ae* is the random additive genetic-by-environment interaction effect with ae~ $N\left(0,\sigma_{ae}^{2}⊗I\right)$; r is the random block effect nested in the trial r~ $N\left(0,\sigma_{r(t)}^{2}⊗I\right),$ where t is the t^th^ progeny test; row and column are the nested random row/column effect in each test with row~$\;N\left(0,\sigma_{row(t)}^{2}⊗I\right)$ and column ~$\;N\left(0,\sigma_{column(t)}^{2}⊗I\right)$ respectively. Z_1_ to Z_5_ denote the incidence design matrices relating following random effects *a*, *ae*, *r*, *row*, and *column*, respectively, to observations *Y*. *Post hoc* factors were listed in the Table 2. The nearest neighbor principal components are the fixed covariate effects in both the single-site analyses and MET. The total variance matrix can be partitioned into components based on the vectors of random effects mentioned previously as follows,

$$V=\sigma_{A}^{2}{Z_{1}AZ}_{1}^{'}+\sigma_{ae}^{2}{Z_{2}Z}_{2}^{'}{+\sigma }_{rt}^{2}{Z_{3}Z}_{3}^{'}+\sigma_{row(t)}^{2}{Z_{4}Z}_{4}^{'}+\sigma_{column(t)}^{2}{Z_{5}Z}_{5}^{'}+R$$ (2)

The best linear unbiased estimation (BLUE) of fixed effect (*β*) and best linear unbiased prediction (BLUP) of random effects (*a*, *r*, *ae*, *row*, *column*) are solutions to the following mixed model equations,

$$\left[\begin{array}{c} \hat{\beta }\\ \hat{a}\\ \hat{\begin{array}{c} ae\\ \hat{rt} \end{array}}\\ \hat{row}\\ \hat{column} \end{array}\right]\;={\left[\begin{array}{cccccc} I^{\prime }I & I\prime Z_{1} & I\prime Z_{2} & I\prime Z_{3} & I\prime Z_{4} & I\prime Z_{5}\\ Z_{1}\prime I & Z_{1}\prime Z_{1}+A^{-1}\frac{{\hat{\;\sigma_{E}}}^{2}}{{\hat{\;\sigma_{A}}}^{2}} & Z_{1}\prime Z_{2} & Z_{1}\prime Z_{3} & Z_{1}\prime Z_{4} & Z_{1}\prime Z_{5}\\ Z_{2}\prime I & Z_{2}\prime Z_{1} & Z_{2}\prime Z_{2}+I_{ae}\frac{{\hat{\;\sigma_{E}}}^{2}}{{\hat{\;\sigma_{AE}}}^{2}} & Z_{2}\prime Z_{3} & Z_{2}\prime Z_{4} & Z_{2}\prime Z_{5}\\ Z_{3}\prime I & Z_{3}\prime Z_{1} & Z_{3}\prime Z_{2} & Z_{3}\prime Z_{3}+I_{rt}\frac{{\hat{\;\sigma_{E}}}^{2}}{\hat{\sigma_{rt}^{2}}} & Z_{3}\prime Z_{4} & Z_{3}\prime Z_{5}\\ Z_{4}\prime I & Z_{4}\prime Z_{1} & Z_{4}\prime Z_{2} & Z_{4}\prime Z_{3} & Z_{4}\prime Z_{4}+I_{row}\frac{{\hat{\;\sigma_{E}}}^{2}}{\hat{\sigma_{row\left(t\right)}^{2}}} & Z_{4}\prime Z_{5}\\ Z_{5}\prime I & Z_{5}\prime Z_{1} & Z_{5}\prime Z_{2} & Z_{5}\prime Z_{3} & Z_{5}\prime Z_{4} & Z_{5}\prime Z_{5}+I_{column}\frac{{\hat{\;\sigma_{E}}}^{2}}{\hat{\sigma_{column\left(t\right)}^{2}}} \end{array}\right]}^{-1}\left[\begin{array}{c} I\prime Y\\ Z_{1}\prime Y\\ Z_{2}\prime Y\\ Z_{3}\prime Y\\ Z_{4}\prime Y\\ Z_{5}\prime Y \end{array}\right]$$ (3)

For MET analyses, except the models with simple residual variance, all other models (heterogeneous variance, and nearest neighbor variance) had two dimensional first-order autoregressive (x and y, AR1) variance of residual given $Cov\left(\varepsilon_{ij},\varepsilon_{ij'}\;\right)=\sigma_{\varepsilon }^{2}Corr\left(\varepsilon_{ij},\varepsilon_{ij'}\;\right)=\sigma_{\varepsilon }^{2}\rho_{j}^{\left|j-j'\right|}\rho_{i}^{\left|i-i'\right|},$ where $\sigma_{\varepsilon }^{2}$ is the residual variance adjusted with no spatial trend; *ε*_*ij*_ is the residual of the individual tree at position (i,j), *ρ* is the correlation coefficient at i-direction (row) or j direction (column). R = $\sigma_{\varepsilon }^{2}AR1+{\;⊕}_{i=1}^{i=n}{\sigma_{e}^{2}}_{i}\;or\;\sigma_{\varepsilon }^{2}AR1+{\sigma_{e}^{2}}_{i}⊗I\;$ [44], where $\sigma_{\varepsilon }^{2}$ AR1 is the variance covariance matrix of the spatial residual variance; AR1 is *Σ*_*row*_ ⊗ *Σ*_*column*_ containing the correlation coefficients *ρ*, where *Σ*_*row*_ and *Σ*_*column*_ are the row and column correlation matrices [46]; for the heterogeneous variances model, the independent residual variance-covariance matrix is as ${⊕}_{i=1}^{i=n}{\sigma_{e}^{2}}_{i}$; and R, i, n, are the variance-covariance matrix of the random effects, i^th^ index of trial, the total number of trials; the simple residual variance-covariance is as $\sigma_{e}^{2}⊗I,$ where $\sigma_{e}^{2}$ denotes the residual variance within trial and residual e~*MVN*(0,*R*).

After the pre-assessment of genetic parameters of single sites, the AFC series 1 and TFS series 2 sites were dropped from the MET analysis due to the low genetic control for selection potential in the MET. Thus, only four tests were reported in the MET results, with two series and three states still covered by the four trials. The single-site analyses covered all six trials with the *post hoc* adjustments. The variance structure and details of predictors are listed in Table 2. We also ran the full original RCBD model that was as following with random effects for comparing with the baseline parameters of variances

$$Y=X\beta +Z_{1}a+Z_{2}ae+Z_{3}r+Z_{4}\;series+Z_{5}Provenance+\;e$$ (4)

where, the fixed effect is only the intercept; series are the two series of the field tests; provenance is the provenance groups (Arkansas, Texas, and Mississippi) of the families; trial effects are nested within each series. Combining both series could increase the capacity for ranking families across all series by adjusting the series effect. The variance components of the series were significantly different from zero for HT, DBH, and volume but the magnitudes were negligible (<0.001). The variance components were from 6% to 40% of those of series for all traits except survival which was not significantly different from zero. Thus, the main models excluded both factors of series and provenances.

### Details of all six sub-blocking methods addressing the main post hoc sources

1. Simple residual variance (SUBBLOCKING) model applied the sub-blocking (random effects) with the 1st order autoregressive variance of residual;
2. Heterogeneous residual (SUBHETERO) model used the sub-blocking (random effects) and the 1st order autoregressive variance of residual plus the heterogeneous variance-covariance structure for the spatial independent residual;
3. Nearest neighbor residual covariance with simple residual variance (SUBNN) model employed sub-blocking (random effects), competition index based on the nearest number of trees (fixed effects), and the 1st order autoregressive residual variance;
4. Nearest neighbor distance with heterogeneous residual variance (SUBNNHETERO) model used the sub-blocking (random effects), competition index based on the nearest number of trees (fixed effects), and the 1st order autoregressive variance of residual plus the heterogeneous variance-covariance structure for the spatial independent residual;
5. Nearest neighbor distance covariate with heterogeneous residual variance (SUBNNPCA1) model employed sub-blocking (fixed effects), principal components 1–3 based on the distance matrix (fixed effects), and the 1st order autoregressive variance of residual;
6. Combined nearest neighbor distance covariate with simple residual variance (SUBNNPCA2) model used sub-blocking (fixed effects), competition index based on the nearest number of trees (fixed effects), principal components 1–3 based on the distance matrix (fixed effects), and the simple variance of residual;

### Details of all six complete blocking methods addressing the main post hoc sources

7. Simple residual variance (BLOCKING) model uses the complete blocking (fixed effects) with the 1st order autoregressive variance of residual;
8. Heterogeneous residual variance (BLKHETERO): the complete blocking (fixed effects) and the 1st order autoregressive variance of residual plus the heterogeneous variance-covariance structure for the spatial independent residual;
9. Nearest neighbor covariate with simple residual variance (BLKNN): the complete blocking (fixed effects), competition index based on the nearest number of trees (fixed effects), and the 1st order autoregressive variance of residual;
10. Nearest neighbor distance covariate with heterogeneous residual variance (BLKNNHETERO): the complete blocking (random effects), competition index based on the nearest number of trees (fixed effects), and the 1st order autoregressive variance of residual plus the heterogeneous variance-covariance structure for the spatial independent residual;
11. Nearest neighbor combined covariate with heterogeneous residual variance (NNPCA1): the complete blocking (fixed effects), principal components 1–3 based on the distance matrix (fixed effects), and the 1st order autoregressive variance of residual;
12. Combined nearest neighbor and distance covariates with simple residual variance (NNPCA2): the complete blocking (fixed effects) competition index based on the nearest number of trees (fixed effects), principal components 1–3 based on the distance matrix (fixed effects), and the simple variance of residual; the trial was all fitted as the fixed effect in all the twelve models for the multi-environment tests and details of the model structure were listed previously.

### Quantitative genetic parameter calculations

We estimated the narrow-sense heritability for multiple site analyses as

$$\hat{h_{}^{2}}=\frac{\hat{\sigma_{A}^{2}}}{\hat{\sigma_{P}^{2}}}=\frac{\hat{\sigma_{A}^{2}}}{\left(\hat{\sigma_{A}^{2}}+\hat{\sigma_{AE}^{2}}+\hat{\sigma_{E}^{2}}\right)}$$ (5)

where, $\hat{\sigma_{A}^{2}}$ is the additive genetic variance component based on the individual tree model [1, 4]; $\hat{\sigma_{AE\;}^{2}}$ is the additive genetic by site interaction variance component (GxE); $\hat{\sigma_{E}^{2}}$ is the residual environment variance for models with a simple variance; the among-trial average of residual variance is used for the heterogeneous residual model; $\hat{\sigma_{P}^{2}}$ is the phenotypic variance component represented by the sum of $\hat{\sigma_{A}^{2}},\;\hat{\sigma_{AE}^{2}}$ and $\hat{\sigma_{E}^{2}}$.

Type-B genetic correlation was estimated as follows

$${\hat{r}}_{b}=\frac{\hat{\sigma_{A}^{2}}}{\left(\hat{\sigma_{A}^{2}}+\hat{\sigma_{AE}^{2}}\right)}$$ (6)

Coefficients of variation (CV) were calculated as follows including the additive genetic CV_A_, phenotypic CV_P_, and random environment CV_E_,

$${\hat{CV}}_{A}=\frac{\hat{\sigma_{A}^{2}}}{\overset{-}{\text{phenotype}}};\;{\hat{CV}}_{P}=\frac{\hat{\sigma_{P}^{2}}}{\overset{-}{\text{phenotype}}};\;{\hat{CV}}_{E}=\frac{\hat{\sigma_{E}^{2}}}{\overset{-}{\text{phenotype}}}$$ (7)

where, $\overset{-}{\text{phenotype}}$ is the trait mean.

Accuracies of breeding values were calculated as follows

$$r_{i}=\sqrt[{}]{1-\frac{{se}^{2}}{\left(1+f_{i}\right)*\hat{\sigma_{A}^{2}}}}$$ (8)

where, se^2^ and f_i_ were the prediction error variance and the inbreeding coefficient of i^th^ individual tree, respectively. We used ASReml-R v3.0 [47] to fit the genetic models, and the standard error of variance components (e.g., heritabilities, type-B genetic correlations, and CVs) were calculated with the delta method [48]. The correlations of breeding values among sites were calculated as the Pearson’s correlation coefficients of breeding values of growth traits versus longitude and latitude of provenance locations with modified *chart*.*correlation* of the R package *PerformanceAnalytics*. Other bar charts were constructed with the *ggplot2* package in R. Map data were provided from the *urbnmapr* package in R.

## Results

### Phenotypic variation, genetic parameters of growth traits

#### Multi-environment test (MET)

The additive genetic by site interaction variance (GxE) was not significantly different from zero or estimable for most of the traits (Table 3). Type-B genetic correlations (r_B_) of height were 0.9±0.2~1.0±<0.01 demonstrating little to no additive genetic-by-environment interaction (GxE) effect (S1 Fig, Table 3). The stability of families was high within the breeding regions in terms of growth performance though extensive field tests are necessary to further validate the r_B_. Though two series were combined in the same mixed model, there was low GxE interaction expressed because two of the three pairs of tests from two series were established at adjacent locations. We found no divergence of the breeding values rankings of parents and individual trees among different BLUP models for height (S7 Fig).

**Table 3:** Genetic parameters of different modeling results for height, DBH, volume, and survival traits.

Parameter	RCBD	SUBB	SUBH	SUBNN	SUBNNH	SUBNNPCA1	SUBNNPCA2	BLOCKING	BLKH	BLKNN	BLKNNH	NNPCA1	NNPCA2
	**Height**												
V_A_	0.405 (0.129)	0.407 (0.127)	0.401 (0.126)	0.403 (0.136)	0.398 (0.134)	0.385 (0.123)	0.390 (0.133)	0.414 (0.130)	0.417 (0.130)	0.414 (0.130)	0.414 (0.130)	0.395 (0.126)	0.395 (0.126)
V_AE_	0 (0)	0 (NA)	0 (0)	0.006 (0.078)	0.003 (0.077)	0 (0)	0.003 (0.077)	0 (0)	0 (0)	0 (NA)	0 (0)	0 (0)	0 (NA)
V_E_	2.315 (0.098)	1.995 (0.102)	1.990 (0.105)	2.002 (0.105)	1.997 (0.108)	1.986 (0.103)	2.000 (0.104)	2.290 (0.098)	2.288 (0.1)	2.269 (0.097)	2.259 (0.100)	2.272 (0.098)	2.263 (0.096)
V_P_	2.721 (0.093)	2.402 (0.098)	2.391 (0.102)	2.411 (0.098)	2.398 (0.102)	2.155 (0.100)	2.393 (0.097)	2.704 (0.093)	2.705 (0.097)	2.683 (0.092)	2.674 (0.096)	2.667 (0.095)	2.659 (0.091)
h^2^	0.149 (0.044)	0.170 (0.049)	0.168 (0.049)	0.167 (0.053)	0.166 (0.053)	0.162 (0.048)	0.163 (0.052)	0.153 (0.045)	0.154 (0.045)	0.154 (0.045)	0.155 (0.045)	0.148 (0.044)	0.149 (0.044)
	**DBH**												
V_A_	0.746 (0.312)	0.751 (0.350)	0.793 (0.363)	0.727 (0.350)	0.770 (0.365)	0.765 (0.354)	0.704 (0.343)	0.756 (0.349)	0.782 (0.36)	0.730 (0.349)	0.756 (0.361)	0.746 (0.351)	0.699 (0.341)
V_AE_	0 (0)	0.036 (0.299)	0.028 (0.301)	0.077 (0.307)	0.072 (0.310)	0.019 (0.298)	0.070 (0.305)	0.008 (0.296)	0.013 (0.299)	0.049 (0.304)	0.058 (0.308)	0.014 (0.298)	0.055 (0.303)
V_E_	9.289 (0.321)	8.666 (0.381)	9.163 (0.409)	8.690 (0.382)	9.188 (0.411)	9.203 (0.408)	8.706 (0.381)	9.264 (0.340)	9.657 (0.371)	9.256 (0.341)	9.649 (0.372)	9.64 (0.368)	9.245 (0.339)
V_P_	10.035 (0.302)	9.452 (0.346)	9.983 (0.383)	9.494 (0.348)	10.03 (0.384)	9.511 (0.419)	9.480 (0.346)	10.028 (0.304)	10.451 (0.344)	10.035 (0.305)	10.462 (0.344)	10.400 (0.341)	9.998 (0.303)
h^2^	0.074 (0.030)	0.079 (0.036)	0.079 (0.036)	0.077 (0.036)	0.077 (0.036)	0.077 (0.035)	0.074 (0.035)	0.075 (0.034)	0.075 (0.034)	0.073 (0.034)	0.072 (0.034)	0.072 (0.033)	0.070 (0.033)
	**Volume**												
V_A_	0.085 (0.036)	0.089 (0.037)	0.088 (0.035)	0.089 (0.037)	0.087 (0.035)	0.085 (0.034)	0.087 (0.036)	0.087 (0.036)	0.088 (0.035)	0.086 (0.036)	0.088 (0.035)	0.084 (0.034)	0.084 (0.036)
V_AE_	0 (0)	0 (NA)	0 (0)	0 (NA)	0 (0)	0 (0)	0 (NA)	0 (0)	0 (0)	0 (NA)	0 (0)	0 (0)	0 (NA)
V_E_	1.116 (0.038)	1.016 (0.043)	1.144 (0.048)	1.019 (0.043)	1.149 (0.048)	1.146 (0.047)	1.019 (0.043)	1.113 (0.038)	1.184 (0.043)	1.115 (0.038)	1.185 (0.043)	1.183 (0.043)	1.111 (0.038)
V_P_	1.201 (0.036)	1.106 (0.041)	1.232 (0.046)	1.108 (0.041)	1.237 (0.046)	1.160 (0.045)	1.106 (0.04)	1.200 (0.036)	1.272 (0.042)	1.201 (0.036)	1.272 (0.042)	1.268 (0.042)	1.195 (0.036)
h^2^	0.071 (0.029)	0.081 (0.032)	0.071 (0.028)	0.080 (0.032)	0.071 (0.027)	0.069 (0.027)	0.078 (0.032)	0.072 (0.029)	0.069 (0.027)	0.072 (0.029)	0.069 (0.027)	0.067 (0.026)	0.070 (0.029)
	**Survival**												
V_A_	0.088 (0.139)	0.007 (0.002)	0.007 (0.002)	0.007 (0.002)	0.007 (0.002)	0.007 (0.002)	0.006 (0.002)	0.007 (0.002)	0.008 (0.002)	0.007 (0.002)	0.007 (0.002)	0.007 (0.002)	0.006 (0.002)
V_AE_	0.006 (0.071)	0 (NA)	0 (0)	0 (NA)	0 (0)	0 (0)	0 (NA)	0 (0)	0 (0)	0 (NA)	0 (0)	0 (0)	0 (NA)
V_E_	0.105 (0.070)	0.055 (0.002)	0.064 (0.003)	0.055 (0.002)	0.064 (0.003)	0.064 (0.003)	0.055 (0.002)	0.064 (0.002)	0.069 (0.003)	0.064 (0.002)	0.069 (0.003)	0.069 (0.003)	0.063 (0.002)
V_P_	0.199 (0.070)	0.061 (0.002)	0.071 (0.003)	0.062 (0.002)	0.071 (0.003)	0.066 (0.003)	0.061 (0.002)	0.071 (0.002)	0.077 (0.003)	0.070 (0.002)	0.076 (0.003)	0.076 (0.003)	0.070 (0.002)
h^2^	0.443 (0.570)	0.109 (0.038)	0.105 (0.033)	0.107 (0.037)	0.103 (0.033)	0.102 (0.033)	0.105 (0.037)	0.095 (0.033)	0.099 (0.031)	0.094 (0.033)	0.098 (0.031)	0.096 (0.031)	0.091 (0.033)

Note: Four tests, 2776 trees from 51 families, were included in the analyses; V_A_, additive genetic variance component; V_AE_, additive genetic x environment variance component; V_E_, residual variance component; V_P_, phenotypic variance component; h^2^, narrow-sense heritability;

**RCBD**, is original RCBD model; **SUBB**, incomplete blocking; **SUBH**, incomplete blocking with heterogeneous residual variance; **SUBNN**, incomplete blocking with neighboring effect; **SUBNNH**, incomplete blocking with neighboring effect and heterogeneous residual variance; **SUBNNPCA1**, incomplete blocking with neighboring distance PC model; **SUBNNPCA2**, incomplete blocking with neighboring effect and distance PC model; **BLOCKING**, complete blocking; **BLKH**, complete blocking with heterogeneous residual variance; **BLKNN**, complete blocking with neighboring effect; **BLKNNH**, complete blocking with neighboring effect and heterogeneous residual variance; **NNPCA1**, complete blocking with distance PC1-3 model; **NNPCA2**, complete blocking with neighboring effect and distance PC1-3 model. The RCBD model of survival was not converged.

0 (0), <0.001 (< .001); 0 (NA), < .001 (not available). The narrow-sense heritability of survival is not significantly different from zero (0.443 ±0.570).

The AFC1 and TFS2 tests showed negligible narrow-sense heritability and additive genetic variance and were dropped from the combined MET that included 2,776 trees from 51 families across three breeding regions. Narrow-sense heritabilities and standard errors for height, DBH, volume, and survival were 0.17±0.05, 0.08±0.04, 0.07±0.04, and 0.10±0.03 respectively in the MET of the four tests on average of all models evaluated here (Table 3).

The additive genetic variances were 0.4±0.1, 0.7±0.3, 0.09±0.04, and 0.01±0.002 while the phenotypic variances were 2.5±0.1, 10±0.4, 1.2±0.04, and 0.07±0.002 for height, DBH, volume, and survival respectively (S6 Fig). The additive coefficient of variation (CV_A_) for height and DBH was 6% and 9% and dropped to 0.4% for volume and 0.1% for survival. For the phenotypic coefficients of variation (CV_P_), the estimates were ~32%±1%, 114%±4%, 6%±0.2%, and 0.9%±<0.1% for height, DBH, volume, and survival (S6 Fig).

### Model comparison

Models were compared using the Akaike Information Criterion (AIC) and Bayesian Information Criterion (BIC). AIC was calculated as $AIC=2t-2ln\;(\hat{L})$, where $\;ln\;(\hat{L})$ was log-likelihood of the model and t was the number of variance parameters. BIC was as $BIC=t\;logv-2ln\;(\hat{L})$, where t is the number of variance parameters and v is the residual parameter degrees of freedom [47]. The models with lower AIC and BIC are preferred [49].

**Fig 2:**
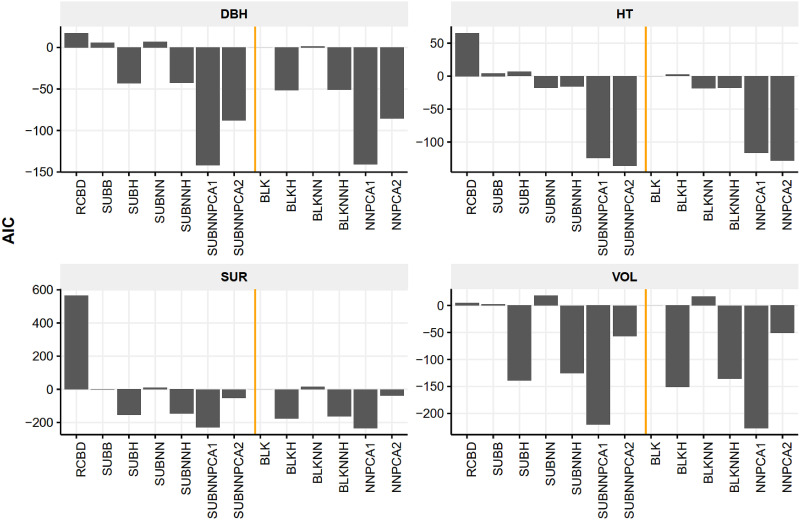
AIC differences of BLUP models of four traits using the complete blocking (BLK) AIC as the benchmark at four selected trials. The negative values indicate more preferred models compared to the BLK model (zero). Note, RCBD, is original RCBD model; SUBB, incomplete blocking; SUBH, incomplete blocking with heterogeneous residual variance; SUBNN, incomplete blocking with neighboring effect; SUBNNH, incomplete blocking with neighboring effect and heterogeneous residual variance; SUBNNPCA1, incomplete blocking with neighboring distance PC model; SUBNNPCA2, incomplete blocking with neighboring effect and distance PC model; BLK, complete blocking; BLKH, complete blocking with heterogeneous residual variance; BLKNN, complete blocking with neighboring effect; BLKNNH, complete blocking with neighboring effect and heterogeneous residual variance; NNPCA1, complete blocking with distance PC model; NNPCA2, complete blocking with neighboring effect and distance PC model. The RCBD model of survival was not converged.

**Fig 3:**
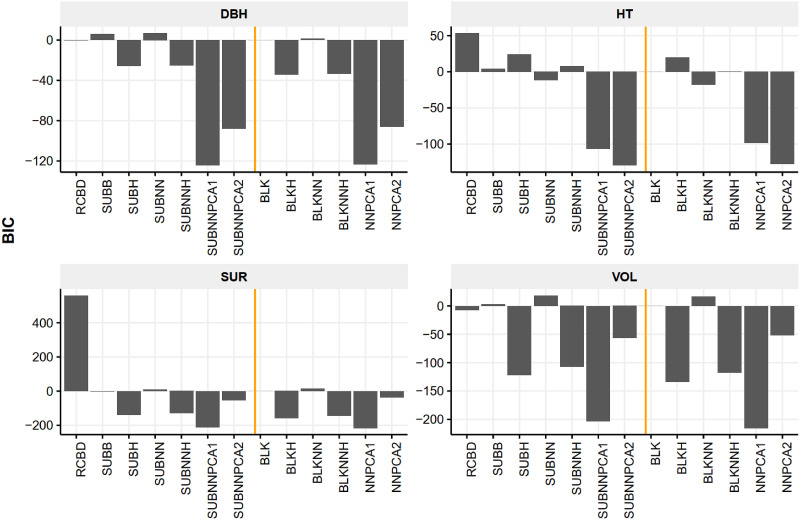
BIC differences of BLUP models of four traits using the complete blocking (BLK) BIC as the benchmark at four selected trials. The negative values indicate more preferred models compared to the BLK model (zero). Note, RCBD, is original RCBD model; SUBB, incomplete blocking; SUBH, incomplete blocking with heterogeneous residual variance; SUBNN, incomplete blocking with neighboring effect; SUBNNH, incomplete blocking with neighboring effect and heterogeneous residual variance; SUBNNPCA1, incomplete blocking with neighboring distance PC model; SUBNNPCA2, incomplete blocking with neighboring effect and distance PC model; BLK, complete blocking; BLKH, complete blocking with heterogeneous residual variance; BLKNN, complete blocking with neighboring effect; BLKNNH, complete blocking with neighboring effect and heterogeneous residual variance; NNPCA1, complete blocking with distance PC model; NNPCA2, complete blocking with neighboring effect and distance PC model. The RCBD model of survival was not converged.

We took the complete blocking (BLK) as the benchmark for AIC (Fig 2) and BIC (Fig 3) comparison by using it as the subtrahend. The original RCBD had the worst fit for survival and height (BIC) and DBH (AIC). The model fitting performance of neighboring methods (SUBNNPCA1 and SUBNNPCA2 as well as the NNPCA1 and NNPCA2) were the preferred models. For height, SUBNNPCA2 and NNPCA2 showed the lowest AIC and BIC, while the methods BLKHETERO, SUBHETERO, and SUBB, SUBNNHETERO were not preferred; for DBH and volume, SUBNNPCA1 and NNPCA1 were preferred due to better fit. The within-group differences of the complete blocking and incomplete blocking methods were higher than the between-group difference in terms of AIC and BIC.

Heritability estimates were improved by 13–14% by using incomplete blocking methods for height, DBH, and volume than the original RCBD method (Table 3). Heritabilities from the original RCBD were lower than other models by 1% to 7% on average among all traits. The heritability differences were about 0–10% between the complete and incomplete blocking methods and the incomplete blocking methods slightly outperform the complete blocking method (Table 3). The RCBD model of survival did not converge. Thus, the genetic parameters of the original RCBD for survival are not significantly different from zero. While heritability estimates were improved via spatial adjustment methods, the accuracies of breeding values among various methods were comparable, ranging from 0.74 to 0.80 for the individual tree breeding values and from 0.80 to 0.95 for the parental breeding values.

### Post hoc adjustment and the variance component estimate of design factors

**Fig 4:**
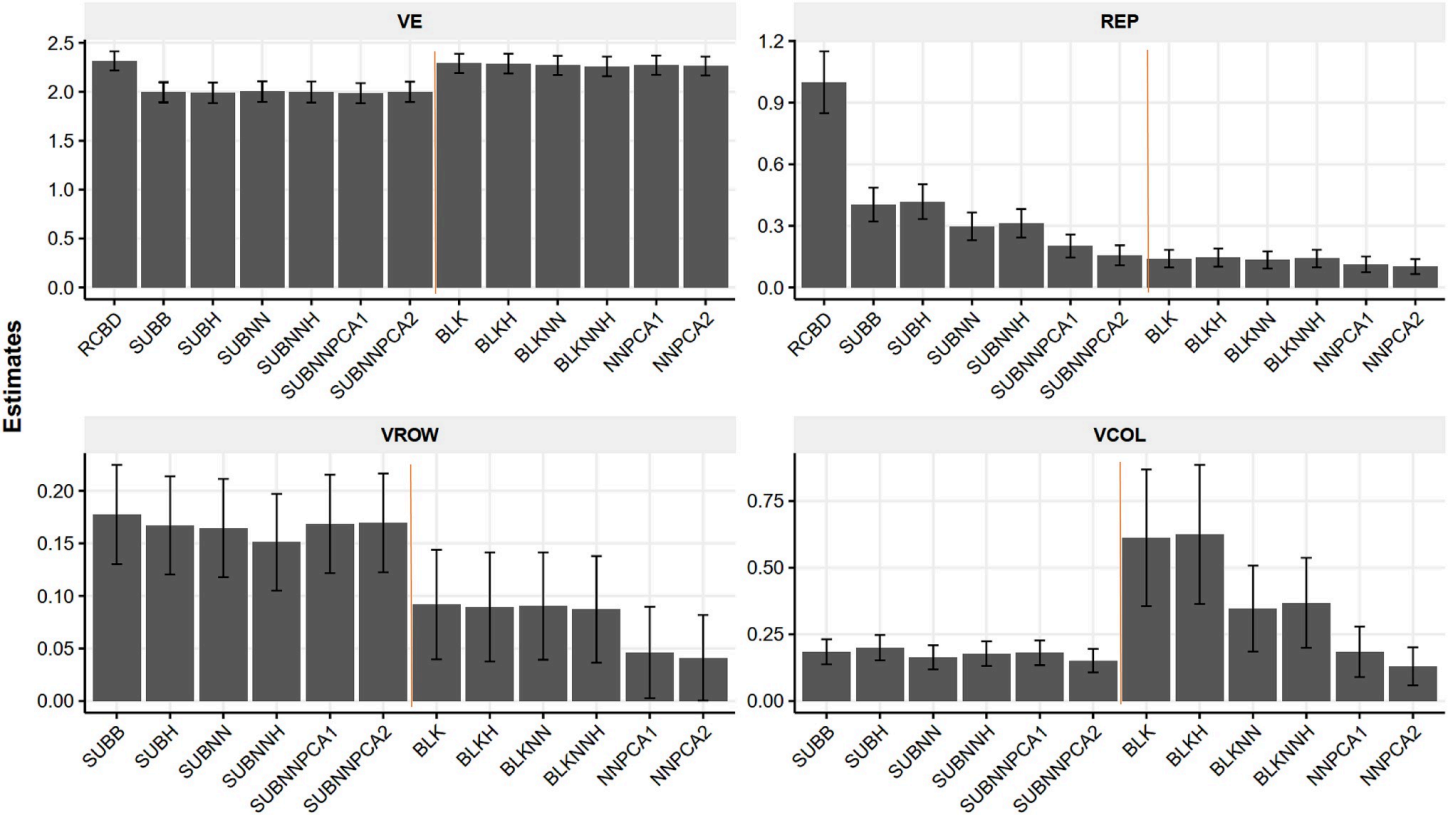
The variance components of height (HT) and the standard errors of several design factors. Note, VE (residual variance), VROW(sub-blocking and complete row blocking), REP (blocking variance), and VCOL (sub-blocking and complete column blocking) for four selected trials. RCBD, is original RCBD model; SUBB, incomplete blocking; SUBH, incomplete blocking with heterogeneous residual variance; SUBNN, incomplete blocking with neighboring effect; SUBNNH, incomplete blocking with neighboring effect and heterogeneous residual variance; SUBNNPCA1, incomplete blocking with neighboring distance PC model; SUBNNPCA2, incomplete blocking with neighboring effect and distance PC model; BLK, complete blocking; BLKH, complete blocking with heterogeneous residual variance; BLKNN, complete blocking with neighboring effect; BLKNNH, complete blocking with neighboring effect and heterogeneous residual variance; NNPCA1, complete blocking with distance PC model; NNPCA2, complete blocking with neighboring effect and distance PC model. The incomplete blocking method reduced HT V_E_ ~14% compared to the original RCBD method (RCBD).

Compared to the RCBD, the incomplete blocking method reduced the residual variance (V_E_) by 14% for HT (Fig 4) while V_E_ of DBH and volume was reduced by 2–4% on average. (S8 Fig). However, the complete blocking showed a similar V_E_ level as the original RCBD result. The residual variance (V_E_) of complete blocking was approximately 10% higher than the counterparts of incomplete blocking methods for HT, DBH, and volume. DBH and volume had a smaller difference in V_COL_ and V_ROW_ between the complete and incomplete blocking methods. (S8 and S9 Figs). For HT, V_ROW_ of the complete blocking models was lower than that of the incomplete blocking methods (Fig 4).

The variance of row for the complete blocking ($\hat{\sigma_{row(t)\;}^{2}}$ or V_ROW_ nested in the trial) ranged from 0.05 to 0.08 (m^2^) for HT and block (i.e.,$\;\hat{\sigma_{rt\;}^{2}}$, V_REP_ nested in the trial) of the complete blocking models ranged from 0.10 to 0.15; both variances were lower than corresponding row/column variances of the incomplete blocking models. (Fig 4). The variance of column ($\hat{\sigma_{column(t)\;}^{2}}\;$ or V_COL_ nested in the trial) ranged from 0.2 to 0.6 (m^2^) and random residual ($\hat{\sigma_{E}^{2}},\;$ V_E_) ranged from 2.0 to 2.3 (m^2^) for HT. Variances from complete blocking were higher than from the incomplete blocking models (Fig 4). For the complete blocking (model BLOCKING) and complete blocking with hetero residual variance model (BLKHETERO), V_COL_ was 3~ 5 times of V_ROW_ and was the most important design variance component of HT following the random residual. Without adjusting the within-block effect or the neighboring effect, the column effect tended to decline when more spatial effects were accounted for (from model BLKNN to NNPCA2, Fig 4).

For the incomplete blocking models, V_ROW_ and V_COL_ were comparable across models for HT indicating the substantial within-block row effect as equivalent to the column effect and the row-column accounted for ~50% of the V_REP_. V_REP_ was more important than other design factors ranging from 0.4 in model SUBB to ~0.2 in SUBNNPCA2. This paralleled the declining trend of V_COL_ in the complete blocking models when more spatial effects were adjusted.

### Weak clinal variation over the landscape

**Fig 5:**
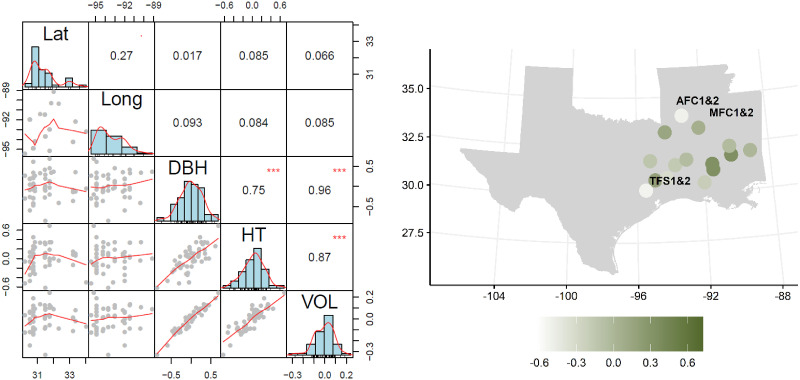
Correlation of provenance of 2^nd^-generation selections (latitude (Lat) and longitude (Long)) versus the breeding values of DBH, HT, and volume, as well as the HT breeding value of 2^nd^-generation parents over the landscape based on the provenance locations and six trials. The green bar indicates the magnitude of volume breeding value, the average of the 12 post hoc methods. The six 2^nd^-generation progeny tests were labeled as (AFC1&2, MFC1&2, and TFS 1&2). Pearson’s correlation coefficients are in the right upper diagonal, the scatter plots are in the lower left diagonal for the pairs of traits, while the diagonal are the histograms of each trait. The p-values are denoted as following ***<0.001, **<0.01, *<0.05. Map data were obtained from *urbnmapr* R package and state boundaries were from the US Census Bureau (www.census.gov).

We found insignificant correlation between the geographic gradients and the breeding values of the 2^nd^ -generation families for HT, DBH, and volume (Fig 5). Those weak correlations suggested no geographical cline of height growth in the region due to the provenance effect; however, fast-growing families tend to originate from the southern region of the study area (i.e., Mississippi) compared to families of lower breeding value from Arkansas and East Texas. The high correlation of 0.87 (p-value <0.0001) between HT and volume demonstrates the potential of using HT alone as a surrogate for selection for volume, a trait that combines DBH and HT. If we fit the quadratic function of HT breeding value (y) as y = u + lat^2^ + long^2^ + lat*long + e, the resultant R^2^ = 0.1436 (p-Value <0.0001). Thus, no quadratic clinal variation along the latitude or longitude for HT was identified.

## Discussion

### Improving environmental variance estimates benefits the genetic parameter estimates

*Post hoc* blocking with incomplete (SUB-) and complete blocking (BLK-) both improved the genetic parameter estimates of *Q*. *pagoda* compared to the original RCBD. Noteworthily, the SUB models outperformed the counterparts of BLK methods. Neighboring effects (e.g., the competition index, neighboring distances, and autoregressive residual variance) were necessary to improve the model fit in both groups of *post hoc* blocking methods. Incomplete blocking has not been frequently tested or applied in genetic tests of *Quercus* species and this study provides variance comparison of multiple model methods grouped into two blocking types in terms of model fitting and genetic parameter estimates.

Our results show that the spatial effect was explicitly partitioned out from the residual variance so that the model fitting and heritability estimates were improved by 1–7% accordingly on average for all growth traits. The V_E_ reduction of height by 14% was slightly lower than previous findings of *Pseudotsuga menziesii* after using the spatial effect adjustment [23]. DBH and volume showed less adjustment of V_E_ than height. When the site preparation and planning are cautious and effective, the spatial effect residing in the V_E_ of the original block tends to decline [36].

The biological reasons for such spatial effect in the typical *Q*. *pagoda* trial are partially due to the root shoot ratio, crown growth characteristics, and shade sensitivity [7]. Thus, shade and competition of neighboring trees tend to pose a strong influence on the tested tree growth in the genetic trial setting. Height and radial growth are linked to advantageous performance in the root growth, which results in increased phenotypic and genetic variation. Results from one field study show that improved trees usually produce roots of greater size compared to the unimproved wild trees [11]. At the individual tree level, the fixed spacing in the testing environment limited the full expansion of the root and crown compared to that of trees regenerated in an open site. Deciduous angiosperms have deeper branching angles (more vertical) than the evergreen gymnosperms and this factor leads to wider crown sizes under the same volume, stem, and branch growth rates in the genetic field tests [8]. After the age 10–15, elevated inter-tree competition effect potentially requires a wider spacing for the fully expression of genetic and phenotypic variances of radial growth in order to accurately evaluate and exploit the genetic gain.

The competition effect demonstrated by the nearest neighbor models using a competition index was associated with the tree spacing and was observed in traits such as survival, diameter, and volume. If the neighboring distance partially contributes to the magnitude of the micro-environment residual, spatial adjustment is necessary to account for the biological characteristics in BLUP.

Column and row, two *post hoc* adjustment factors examined in this study, were determined by the local trial layout. Thus, the differences of row and column were partially due to the arbitrary mapping and coordinates of trees per block. Other studies following the same manner may have different trends of row-column variance that are dependent on the ordination and geometry of the layout. If the two directions are orthogonal regardless of the geographic ordination, the *post hoc* row-column method can be applied [28]. The sum of V_COL_ and V_ROW_ were slightly lower than the V_REP_ of the original RCBD and such trends were associated with incorporated spatial predictors, the fixed effects in the linear mixed models.

### Genetic parameters

Our region-wide investigation of genetic parameters of *Q*. *pagoda* will inform the tree improvement and afforestation program for greater volume and survival for nursery seedling stock and timber production [6], as well as the ecological restoration needs. Previous studies showed that the range of narrow-sense heritability was ~ 0.2–0.3 for volume and 0.2–0.4 for height and diameter at age 15 [9], which was slightly greater than our average estimates of these growth traits, especially for volume and diameter. Our findings warrant moderate genetic control that will ensure selection potential for height growth at age 10, given the similar individual tree heritability reached 0.3 at a site (MFC1) in the Mississippi region.

We could not demonstrate the specific cap value of heritability for the second-generation or earlier selections (< age 10); however, multiple methods resemble similar levels of genetic parameters which were improved by 1%~14% of h^2^ compared to the original RCBD. Low heritability brought an obstacle for breeding specific commercial traits such as DBH and volume. Long-term METs are required for further assessing the genetic parameters before the rotation age. DBH evaluation is promising at a later growing stage while age-10 is sufficient for height selection at high performing sites where crown and floor vegetation are well managed. It is necessary to update the experimental design scheme and layout with more gap size that addresses the horizontal competition of adjacent tested trees in the future, especially for the DBH and volume assessment of *Q*. *pagoda*.

### Quantitative genetic parameters indicated a later age of evaluation for volume selection

Earlier screening of families in genetic trials advances the genetic gain per time unit or breeding cycle [50]. However, this may not be a reality for hardwoods such as *Q*. *pagoda* as previous research suggests efficient genetic evaluation may not occur until as late as age 15 due to the following factors: 1) required crown growth and canopy structure for diameter selection compared to commercial coniferous species [51], such as loblolly pine (*Pinus taeda*) and Douglas-fir (*Pseudotsuga menziesii*) in the genetic test setting, 2) soil requirements, and 3) root growth and competition due to spacing. Maintaining adequate survival may benefit selection as early as age 10 even with larger spacing [9]. The impact of low survival on genetic parameter estimation was evident in one of the trials dropped from this study, TFS2, where there was no adequate genetic control to be estimated.

Poor parameter estimation in genetic trials will limit the full expression of genetic variation and genetic controls for growth under the age 15, or even less than 10 [22], which is the typical evaluation time for commercial trees [23]. In some slow-growing species, including hardwoods, tree diameter growth requires additional evaluation time and gap size management /spacing to be fully expressed in the trial and the within-block variability seems more homogeneous compared to the case for height and survival. Such species are not popularly studied in breeding programs, fewer data source and more knowledge gaps exist and prove problematic for the breeder to benefit from expanding genetic testing and exploiting genetic variation for these species in a timely manner. These bottleneck effects include limit provenance performance over the species range [7]; constrained testing resources for establishing, maintaining, and evaluating broader scales of families and sites; not optimized test design schemes for multiple provenances and breeding zones.

### Model improvement

Our results suggested the incomplete blocking with the *post hoc* methods generated models with improved AIC and BIC, resulting in moderately improved heritabilities compared with results using the original RCBD design, especially for height. The incomplete subblocking with spatial distance adjustment (SUBNNPC1) is preferred due to smaller blocking size and adjustment of neighboring distance, followed by the SUBNNPC2 method which introduced neighboring competition predictors with less payback on AIC/BIC improvement. Subblocking (SUBB) provided greater estimates of heritability. Computation time can be a concern. However, in this study both NNPCA1 and NNPCA2 models converged quickly, probably due to the moderate size of the samples and the number of sites. The converge time for both models were similar, although NNPCA2 took a little bit longer.

The within-blocks sub-blocking method explained untapped environment heterogeneity because the original RCBD blocks potentially violate the homogeneity variance assumption of the linear model [23, 28] and elevate the noise for dissecting the genetic variance. The residual variance estimated with sub-blocking methods was lower when the genetic variance was similar for HT among all models (Fig 4); thus, the heritability of HT was higher by 12% compared to the RCBD.

A previous study also reported that complete blocking with *post hoc* row-column is preferred to adjust the global gradients of the field test [28]; however, in our case, incomplete blocking that reveals the microenvironment heterogeneity is necessary to be considered. There is also some debate about the size of incomplete blocking. We could not test the efficiency (square root(treatment#)) thoroughly here but five trees can provide plausible modeling ability for partitioning variances in this study.

### Tree breeding applications

Standard operating procedures within the Western Gulf Forest Tree Improvement Program (WGFTIP) include progeny test evaluations at ages 5, 10, 15 and 20, with selections made at the earliest ages (5 and 10) to advance gains into the breeding and deployment populations as rapidly as possible. Based on our results the recommended selection age for volume and DBH in *Q*. *pagoda* should be 15 years rather than at age 10, especially in northern sites. This current operational standard confirms with other studies when survival declines from age 10 to 15 years [9]. After the age 10–15, the field evaluation could potentially avoid the random environment effect due to the inter-tree competition. Tree volume is the preferred candidate trait as it encompasses the combination of DBH and height genetic variations.

Seed zones consolidation for volume improvement could be considered due to limited GxE interactions. Although the current breeding region does not address multiple climatic variables, the augmented zone still allows the evaluation of the best families with stable performance. East Louisiana and West Mississippi families are elite candidate pools for volume and height growth which agrees with the previous findings in a smaller regional study [9]. Those families can be relied on for future generation selection and infusing into the breeding population.

While the use of blocking and nearest neighbor distance measurements had limited impacts on the current selection strategies employed by the WGFTIP in its cherrybark oak program over the whole breeding regions, our results do show that use of these techniques *a priori* in the establishment of future tests may allow for earlier selections through the resulting higher heritabilities for selection traits and through the reduction of residual environmental variances responsible for the analytical noise complicating selection decisions.

## Supporting information

S1 Table Genetic parameters of six WGFTIP *Q*. *pagoda* trials before post hoc treatments. (DOCX)

S2 Table The connectivity table showing the shared families tested among six trials. (DOCX)

S1 Fig Type-B genetic correlations with standard errors for height (HT), DBH (DIA), volume (VOL), and survival (SUR) at four selected trials. (DOCX)

S2 Fig Boxplots of breeding values and parental breeding values of height (four selected trials). (DOCX)

S3 Fig Narrow-sense heritabilities of single-site analyses with incomplete blocking. (DOCX)

S4 Fig Narrow-sense heritabilities of single-site analyses with complete blocking. (DOCX)

S5 Fig Narrow-sense heritability and type-B genetic correlation of traits (six trials). (DOCX)

S6 Fig Coefficient of additive genetic variation (CV_A_) and coefficient of phenotypic genetic variation (CV_P_) of three traits (four selected trials). (DOCX)

S7 Fig Boxplots of height breeding values and parental breeding values of MET (four selected trials). (DOCX)

S8 Fig DBH variance components and the standard errors of various design factors. (DOCX)

S9 Fig Volume variance components and the standard errors of several design factors. (DOCX)

S10 Fig Graphical illustration of complete and incomplete experiment designs using a single site as an example. (DOCX)

S1 File Original RCBD analyses method. (DOCX)

S1 Dataset Phenotyping data for growth and survival trait. A data table in comma separated values (CSV) format containing all the measurement data for individual trees at six trials. The first 11 rows describe the variables of the table.
(CSV)
