# Supplementary material for: Post hoc experimental designs improve genetic trial analyses: A case study of cherrybark oak (Quercus pagoda Raf.) genetic evaluation in the western Gulf region, USA
Source: PLoS One. 2023 May 12;18(5):e0285150. doi: 10.1371/journal.pone.0285150 (PMC10180598; doi:10.1371/journal.pone.0285150)
Supplement: S1 Table — (DOCX) [file pone.0285150.s001.docx]

**Supplementary Table 1. Genetic parameters of six WGFTIP *Q. pagoda* trials before post hoc treatments.**

| Parameters | AFC1 | MFC1 | TFS1 | AFC2 | MFC2 | TFS2 |
| --- | --- | --- | --- | --- | --- | --- |
| *Height* |  |  |  |  |  |  |
| V_A_ | <0.001 (NA) | 0.004 (0.002) | 0.003 (0.002) | 0.001 (0.001) | 0<0.001 (0.001) | 0<0.001 (0.001) |
| V_E_ | 0.028 (0.002) | 0.015 (0.001) | 0.039 (0.002) | 0.024 (0.002) | 0.014 (0.001) | 0.015 (0.001) |
| h^2^ | <0.001 (<0.001) | ***0.207 (0.072)*** | 0.080 (0.052) | 0.022 (0.048) | 0.012 (0.070) | 0.018 (0.073) |
| *DBH* |  |  |  |  |  |  |
| V_A_ | <0.001 (NA) | 0.003 (0.002) | 0.003 (0.003) | 0.003 (0.003) | 0<0.001 (0.003) | <0.001 (NA) |
| V_E_ | 0.051 (0.003) | 0.030 (0.002) | 0.050 (0.003) | 0.048 (0.003) | 0.045 (0.004) | 0.037 (0.003) |
| h^2^ | <0.001 (<0.001) | 0.079 (0.050) | 0.060 (0.051) | 0.058 (0.057) | 0.007 (0.064) | <0.001 (<0.001) |
| *Volume* |  |  |  |  |  |  |
| V_A_ | <0.001 (NA) | 0.335 (0.154) | 0.219 (0.144) | 0.179 (0.149) | <0.001 (NA) | <0.001 (NA) |
| V_E_ | 1.780 (0.098) | 2.233 (0.135) | 2.298 (0.144) | 2.330 (0.159) | 3.862 (0.217) | 0.925 (0.063) |
| h^2^ | <0.001 (<0.001) | ***0.131 (0.057)*** | 0.087 (0.055) | 0.071 (0.058) | <0.001 (<0.001) | <0.001 (<0.001) |
