# Supplementary material for: Post hoc experimental designs improve genetic trial analyses: A case study of cherrybark oak (Quercus pagoda Raf.) genetic evaluation in the western Gulf region, USA
Source: PLoS One. 2023 May 12;18(5):e0285150. doi: 10.1371/journal.pone.0285150 (PMC10180598; doi:10.1371/journal.pone.0285150)
Supplement: S2 Table — (DOCX) [file pone.0285150.s002.docx]

**Supplementary Table 12 The connectivity table showing the shared families tested among six trials**

|  | 1. AFC1 | 1. AFC2 | 1. MFC1 | 1. MFC2 | 1. TFS1 | 1. TFS2 |
| --- | --- | --- | --- | --- | --- | --- |
| AFC1 | 29 | 0.52 | 0.66 | 0.72 | 0.86 | 0.52 |
| AFC2 | *15* | 29 | 0.62 | 0.69 | 0.62 | 0.62 |
| MFC1 | *19* | *18* | 34 | 0.65 | 0.56 | 0.56 |
| MFC2 | *21* | *20* | *22* | 36 | 0.67 | 0.61 |
| TFS1 | *25* | *18* | *19* | *24* | 32 | 0.59 |
| TFS2 | *15* | *18* | *19* | *22* | *19* | 31 |

Note, the diagonal is the number of families tested per site; the upper diagonal showed the ratio of common families against the total families of the previous trial; the lower diagonal is the number of common families between two tests in both vertical and horizontal directions. There are 21 families (62%) tested in both series.
