# Supplementary material for: Post hoc experimental designs improve genetic trial analyses: A case study of cherrybark oak (Quercus pagoda Raf.) genetic evaluation in the western Gulf region, USA
Source: PLoS One. 2023 May 12;18(5):e0285150. doi: 10.1371/journal.pone.0285150 (PMC10180598; doi:10.1371/journal.pone.0285150)
Supplement: S2 Fig — (DOCX) [file pone.0285150.s004.docx]

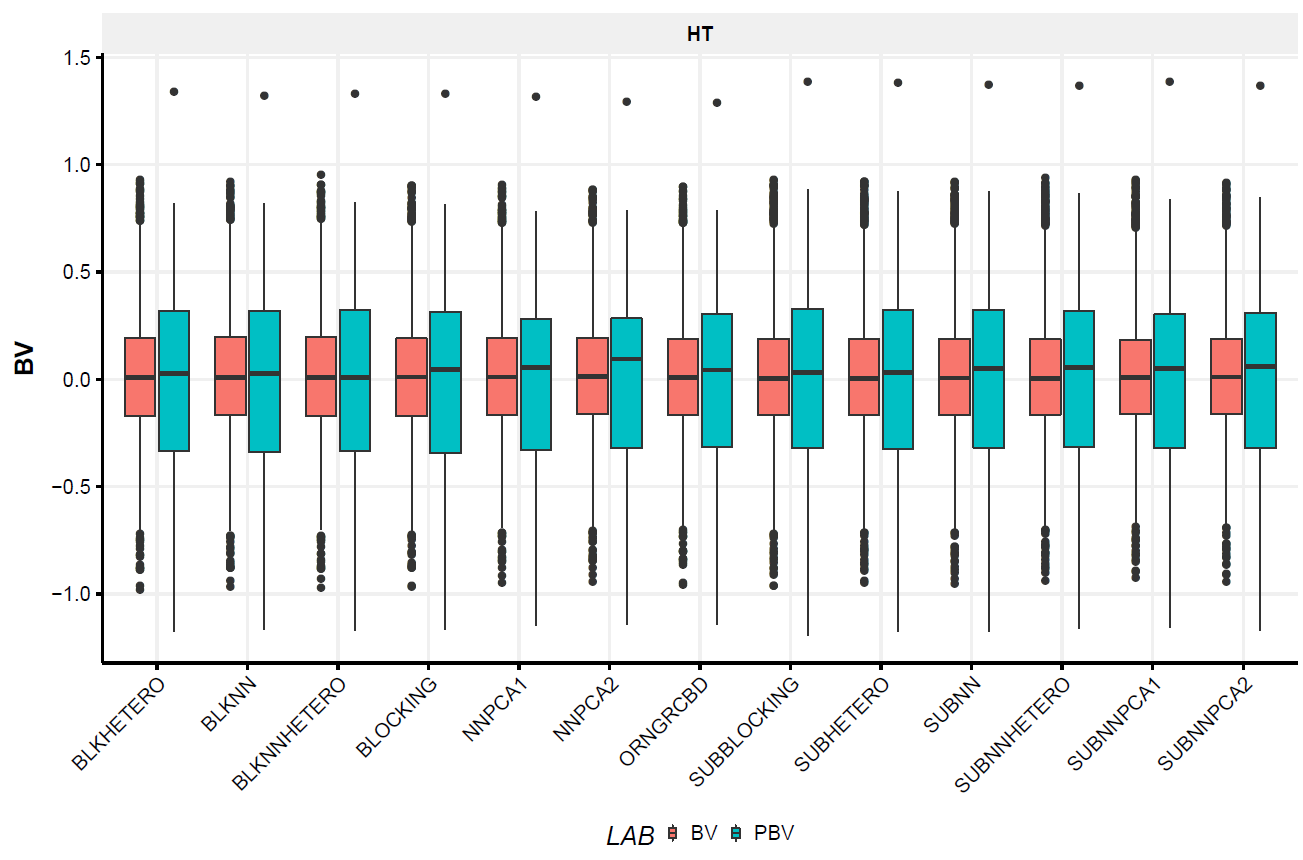


Supplementary Figure 3 Boxplots of breeding values and parental breeding values of height (four selected trials)
