# Supplementary material for: Post hoc experimental designs improve genetic trial analyses: A case study of cherrybark oak (Quercus pagoda Raf.) genetic evaluation in the western Gulf region, USA
Source: PLoS One. 2023 May 12;18(5):e0285150. doi: 10.1371/journal.pone.0285150 (PMC10180598; doi:10.1371/journal.pone.0285150)
Supplement: S3 Fig — (DOCX) [file pone.0285150.s005.docx]

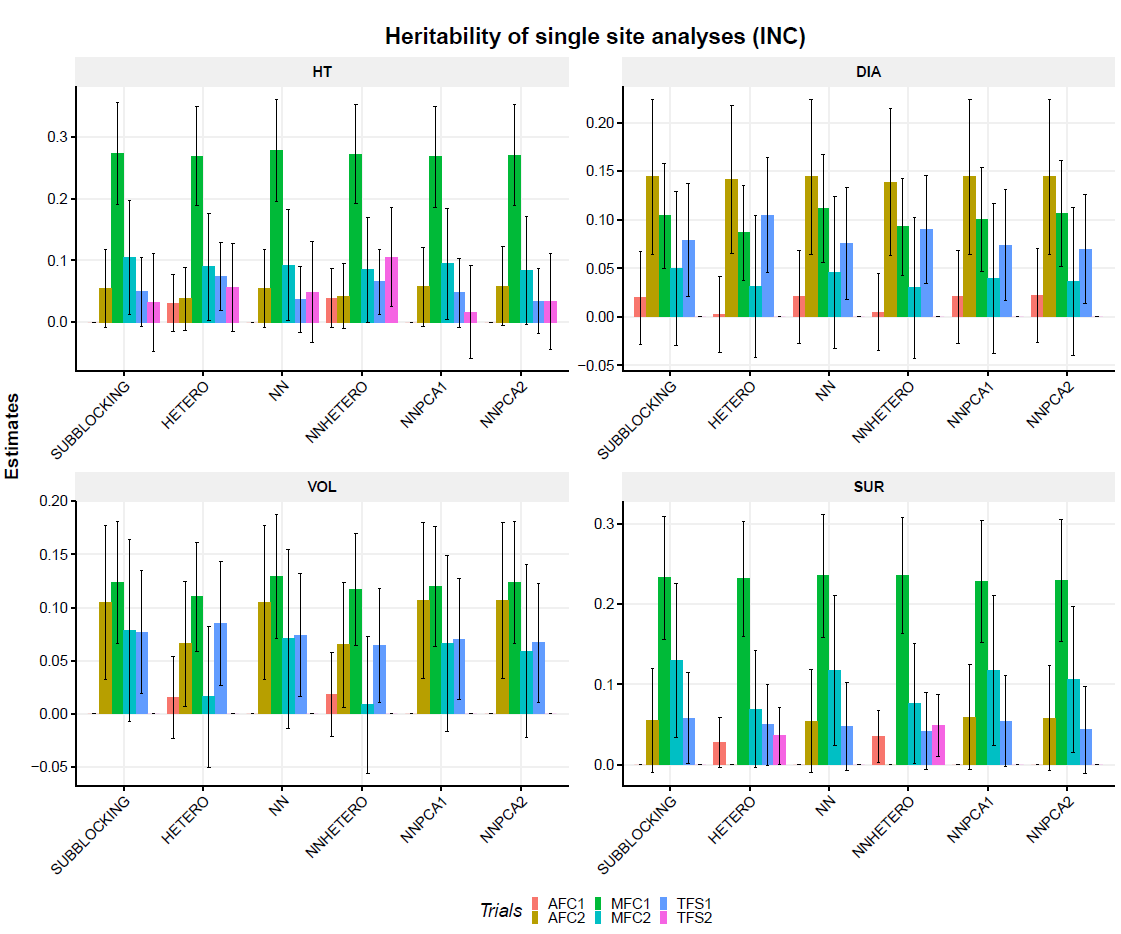


**Supplementary Figure 4. Narrow-sense heritabilities of single-site analyses with incomplete blocking**.

SUBBLOCKING, the incomplete blocking; HETERO, incomplete sub-blocking with heterogeneous variance; NN, nearest neighbor using competition index; NNHETRO competition index with heterogeneous variance model; SUBNNPCA1, incomplete blocking with distance PC model; SUBNNPCA2, incomplete blocking with neighboring effect and distance PC model
