# Supplementary material for: Post hoc experimental designs improve genetic trial analyses: A case study of cherrybark oak (Quercus pagoda Raf.) genetic evaluation in the western Gulf region, USA
Source: PLoS One. 2023 May 12;18(5):e0285150. doi: 10.1371/journal.pone.0285150 (PMC10180598; doi:10.1371/journal.pone.0285150)
Supplement: S5 Fig — (DOCX) [file pone.0285150.s007.docx]

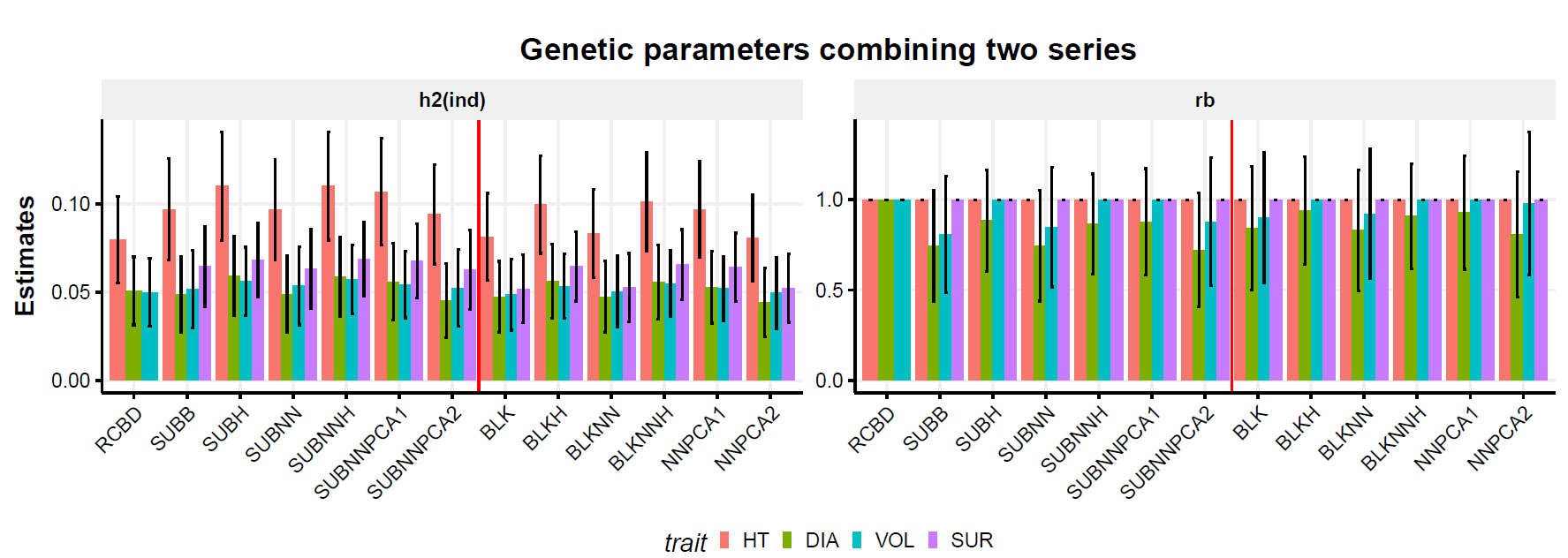


Supplementary Figure 6 Narrow-sense heritability and type-B genetic correlation of traits (six trials)
