# Supplementary material for: Post hoc experimental designs improve genetic trial analyses: A case study of cherrybark oak (Quercus pagoda Raf.) genetic evaluation in the western Gulf region, USA
Source: PLoS One. 2023 May 12;18(5):e0285150. doi: 10.1371/journal.pone.0285150 (PMC10180598; doi:10.1371/journal.pone.0285150)
Supplement: S9 Fig — (DOCX) [file pone.0285150.s011.docx]

**Supplementary Figure 10.**


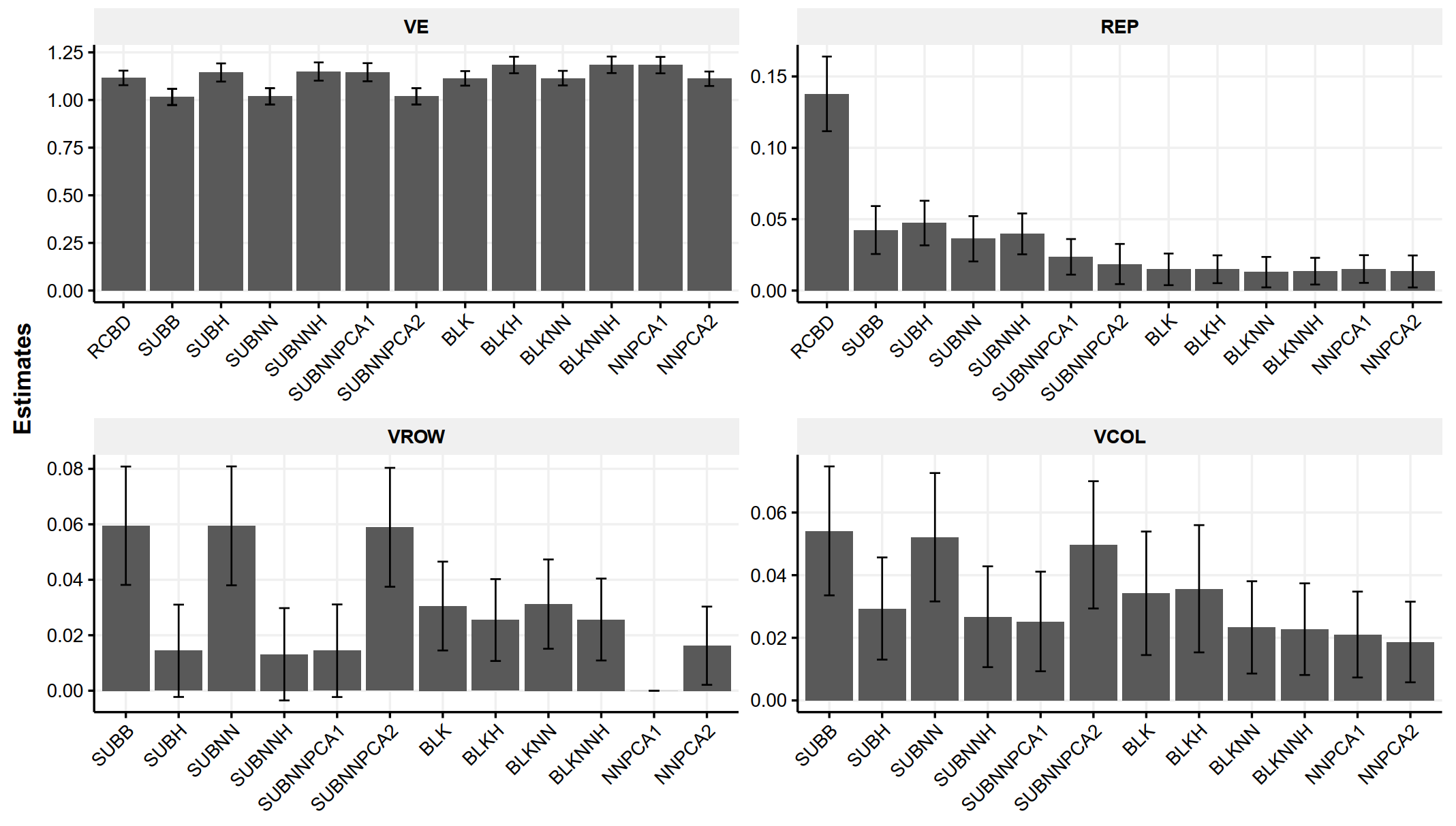


**Supplementary Figure 10. Volume variance components and the standard errors of several design factors.**

Note, VE (residual variance), VROW (sub-blocking and complete row blocking), REP (blocking variance), and VCOL (sub-blocking and complete column blocking) for four selected trials. Note, RCBD, is original RCBD model; SUBB, incomplete blocking; SUBH, incomplete blocking with heterogeneous residual variance; SUBNN, incomplete blocking with neighboring effect; SUBNNH, incomplete blocking with neighboring effect and heterogeneous residual variance; SUBNNPCA1, incomplete blocking with neighboring distance PC model; SUBNNPCA2, incomplete blocking with neighboring effect and distance PC model; BLK, complete blocking; BLKH, complete blocking with heterogeneous residual variance; BLKNN, complete blocking with neighboring effect; BLKNNH, complete blocking with neighboring effect and heterogeneous residual variance; NNPCA1, complete blocking with distance PC model; NNPCA2, complete blocking with neighboring effect and distance PC model.
