# Supplementary material for: Post hoc experimental designs improve genetic trial analyses: A case study of cherrybark oak (Quercus pagoda Raf.) genetic evaluation in the western Gulf region, USA
Source: PLoS One. 2023 May 12;18(5):e0285150. doi: 10.1371/journal.pone.0285150 (PMC10180598; doi:10.1371/journal.pone.0285150)
Supplement: S10 Fig — (DOCX) [file pone.0285150.s012.docx]

**Supplementary Figure 11**

| 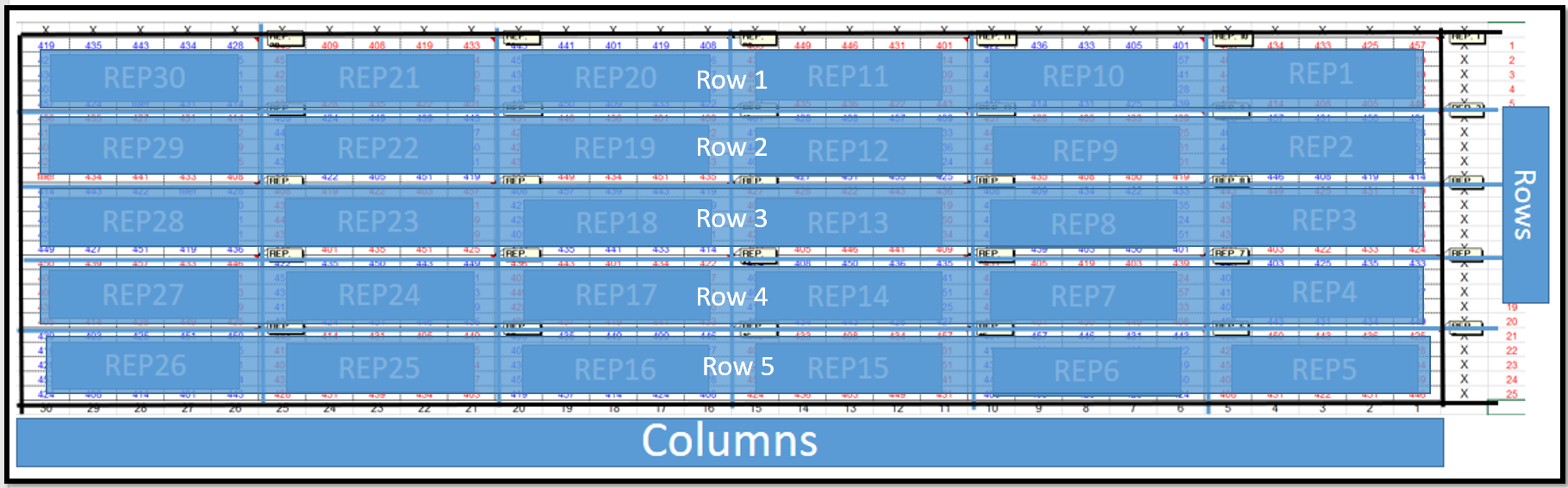  Complete blocking-rows |
| --- |
| 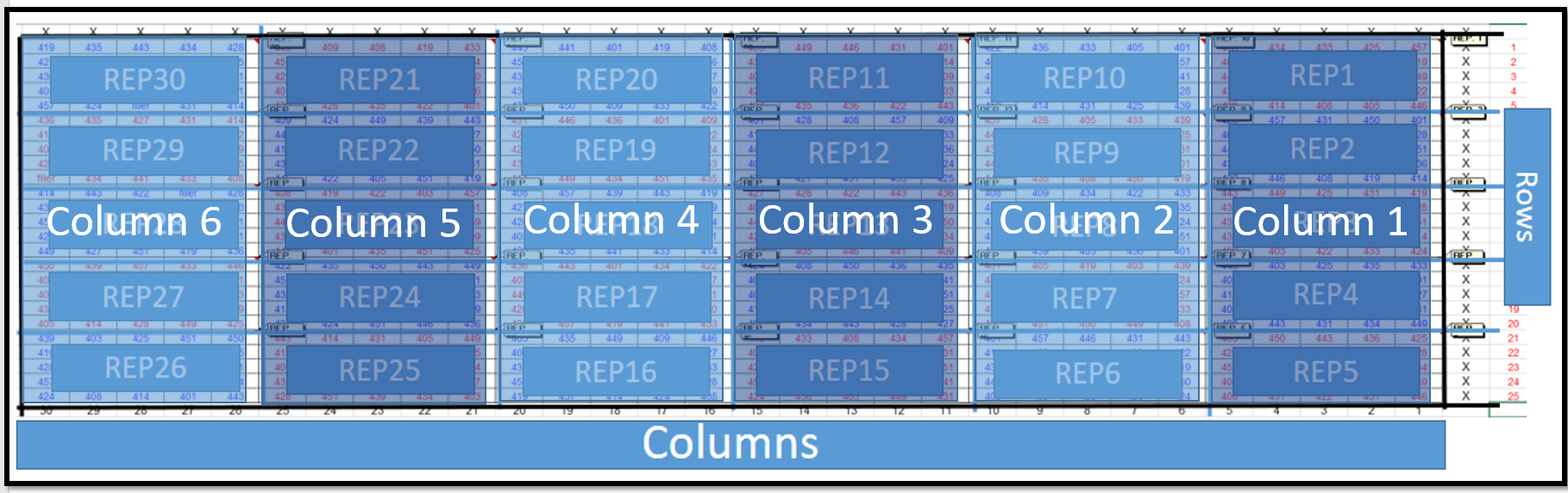  Complete blocking-columns |
| 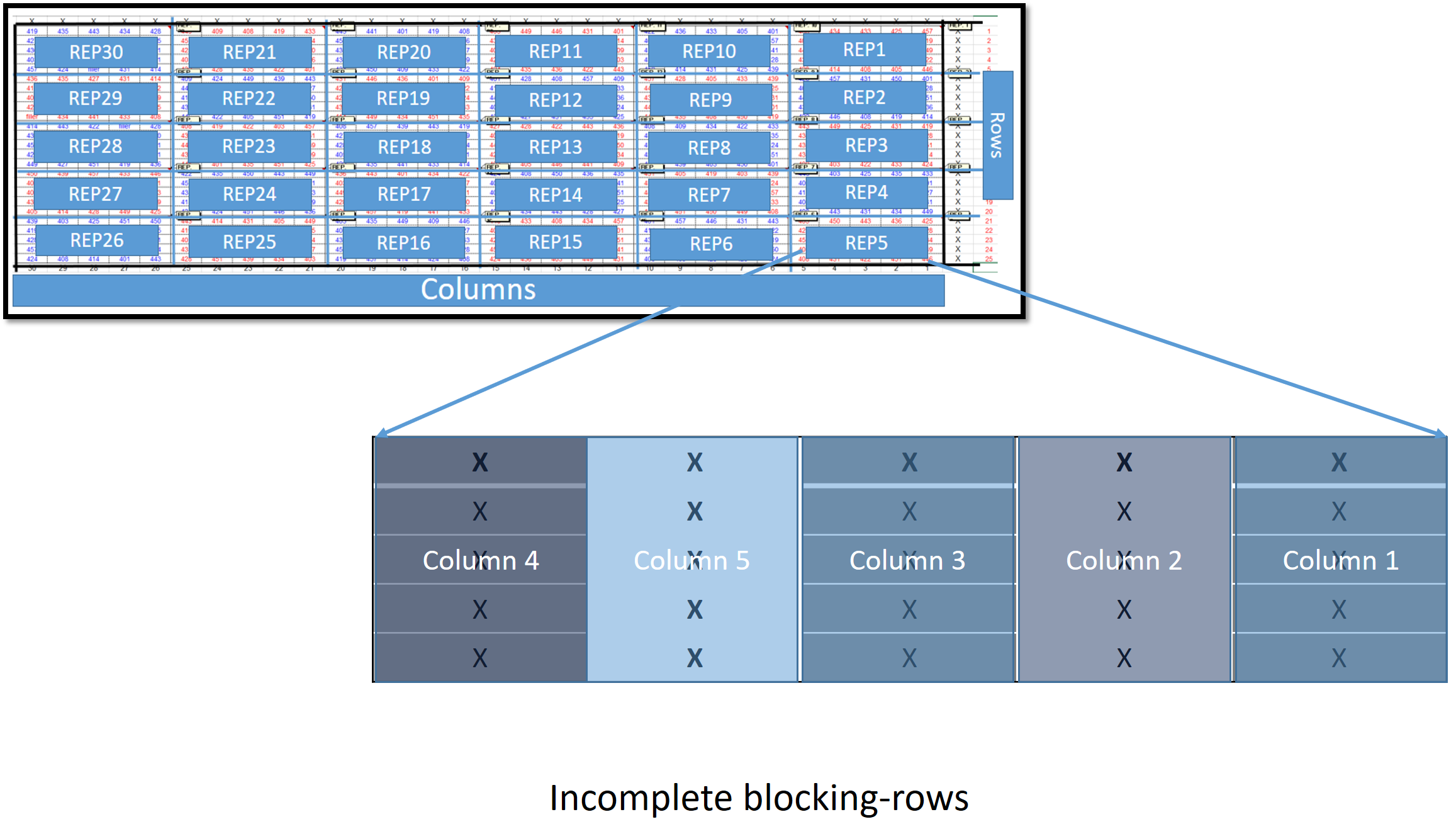Incomplete blocking-columns |
| 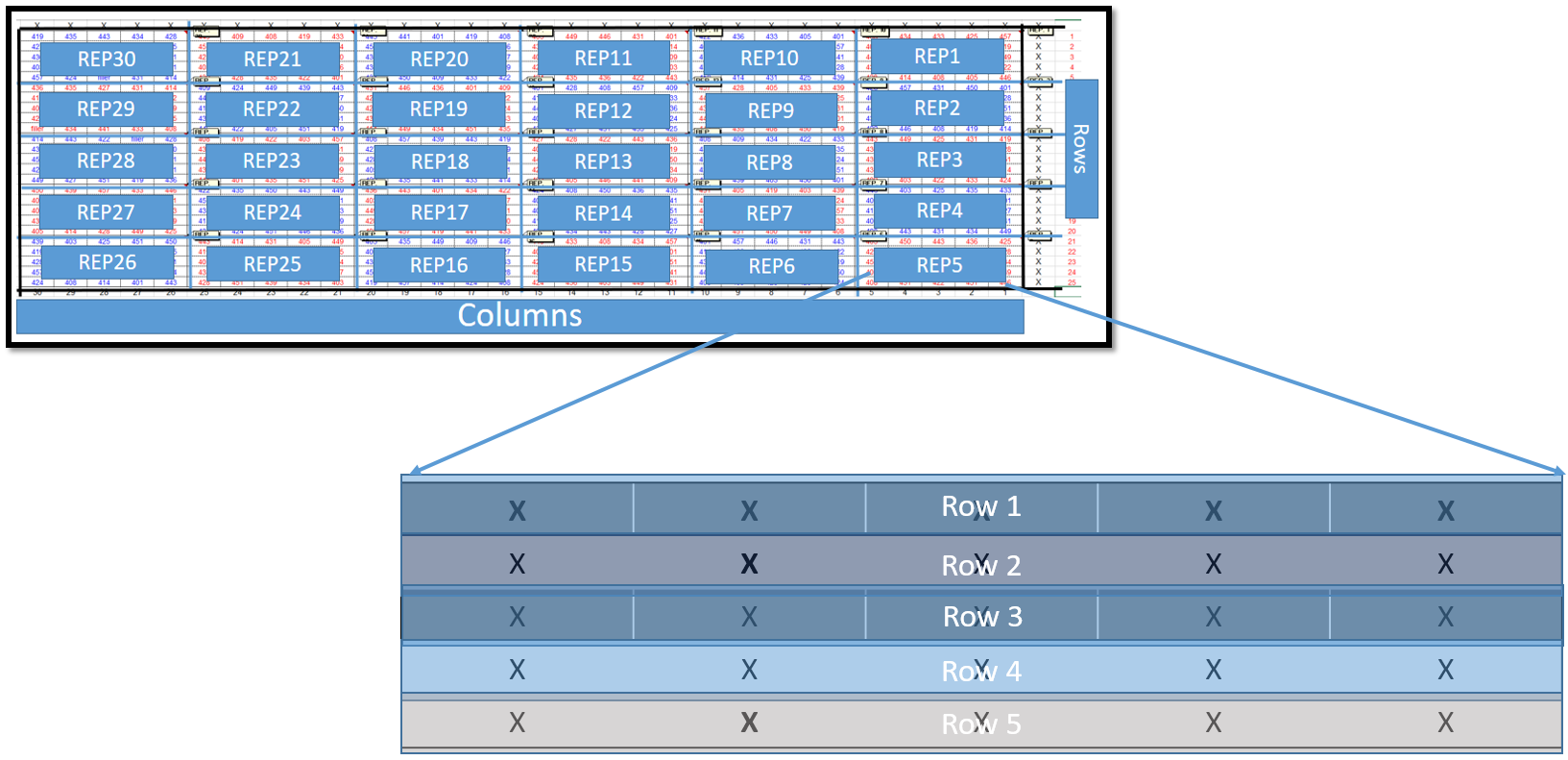  Incomplete blocking-rows |

**Supplementary Figure 11. Graphical illustration of complete and incomplete experiment designs using a single site as an example.**

Note, Reps are the original blocks in the RCBD. Complete blockings grouped adjacent reps into row/columns. The incomplete blocking design delineated the row/column within each rep.
