# Supplementary material for: Post hoc experimental designs improve genetic trial analyses: A case study of cherrybark oak (Quercus pagoda Raf.) genetic evaluation in the western Gulf region, USA
Source: PLoS One. 2023 May 12;18(5):e0285150. doi: 10.1371/journal.pone.0285150 (PMC10180598; doi:10.1371/journal.pone.0285150)
Supplement: S1 File — (DOCX) [file pone.0285150.s013.docx]

# Supporting information

*S13 Original RCBD analyses methods*

Prior to the post hoc blocking analyses, we used the original RCBD design to obtain the baseline of the genetic parameters. The single-site analyses model is

$Y=X\beta+Za+e$

where, y is the vector of the phenotypic measurement of individual tree; $\beta$ is the fixed effect factor of blocks (REP); Z is the incidence design matrix relating the random effect a and the y; $a$ denotes the random vector of half-sib family effects, with a$\sim N(0,\sigma_{A}^{2}A)$, where $\sigma_{A}^{2}$ is the additive genetic variance and A is the pedigree kinship matrix per test; e is the vector of random residuals with e$\sim N\left( 0,\sigma_{e}^{2}I \right)$ where $\sigma_{e}^{2}$ is the variance components corresponding to the random residual. The Y follows the normal distribution $N(X\beta, V)$, and the total variance can be partitioned as follows

$$V=\sigma_{A}^{2}{Z_{1}AZ}_{1}^{'}+ \sigma_{e}^{2}I$$

For the single-site analyses, linear mixed models were applied to partition the fixed block, row-within-block, column-within-block, and random genetic effects for height (HT), diameter at breast height (DBH), volume, and survival using the following equation:

$Y=X\beta+Z_{1}a+Z_{2}r+Z_{3}row+Z_{4}column+e$ [1]

where, **X** is the design matrix of fixed effects to relate **β** to observation Y; **β** is the parameter of fixed effects to be estimated including the x (i.e., row) and y (column) coordinates of each test as continuous predictor defined by the tree location on the layout; **Z_1_ to Z_4_** are the design matrix of random effects based on the model types to relate the random effects including a, r, row blocks, and column blocks. We assume Y follows a normal distribution with the expected value of E(Y)=**Xβ** and the covariance matrix of Var(Y)=V, i.e., Y~MV*N*(X**β, V**); **a** is the vector of random additive genetic effect of female families where a ~ $N\left( 0,\sigma_{A}^{2}\otimes A \right)$, and **A** is the kinship matrix as the previous function; r is the random block effect with r~$N\left( 0,\sigma_{r}^{2}\otimes I \right)$; row and column blocks are the random row/column block effect with row~$N\left( 0,\sigma_{row}^{2}\otimes I \right)$ and column ~$N\left( 0,\sigma_{column}^{2}\otimes I \right)$ respectively; the row and column are defined according to the post hoc block types (i.e., incomplete and modified-complete subblocking ); **I** is the identity matrix of proper size. For the heterogeneous variances model that variance-covariance structure is different from and elaborate compared to the simple residual variance structure, the residual variance is estimated as $R=\oplus_{i=1}^{i=n}{\sigma_{e}^{2}}_{i}$, and R, i, n, are the variance-covariance matrix of random effects, i^th^ index of block, the total number of blocks, respectively; for simple residual variance model, the variance-covariance of residual R = $\sigma_{e}^{2}\otimes I$, and e~$N\left( 0,\sigma_{e}^{2}\otimes I \right)$. Thus, the variance matrix can be partitioned into components of the single-site analyses due to the five vectors of random effects mentioned previously as follows,

$V=\sigma_{A}^{2}{Z_{1}AZ}_{1}^{'}+\sigma_{r}^{2}{Z_{2}Z}_{2}^{'}+\sigma_{row}^{2}{Z_{3}Z}_{3}^{'}+\sigma_{column}^{2}{Z_{4}Z}_{4}^{'}+R$ [2]

Single-site

The single-site analyses showed low to moderate narrow-sense heritability for height (0.00-0.28) that peaked at trial MFC1 (ĥ^2^=0.28), while ĥ^2^ of DBH and volume (0.00-0.15) were lower for both post hoc method groups (Supplementary Figure 4 and 5). Height selection is promising at age 10; and Trial AFC2 showed moderate genetic control for DBH (ĥ^2^ = 0.15). Heritabilities of diameter and volume were estimated at a relatively young age of 10 while height expressed moderate genetic control. Without the post hoc only MFC site showed ĥ^2^>0.1for height, DBH and volume (Supplementary Table 1).
